# Synaptosomal‐Associated Protein 25 kDA (SNAP‐25) Levels in Cerebrospinal Fluid: Implications for Alzheimer's Disease Diagnosis and Monitoring

**DOI:** 10.1002/syn.70010

**Published:** 2025-02-06

**Authors:** Sofia Hjorth Wolner, Helena Sophia Gleerup, Christian Sandøe Musaeus, Peter Høgh, Nicholas J. Ashton, Ann Brinkmalm, Johanna Nilsson, Lana Grötschel, Henrik Zetterberg, Kaj Blennow, Steen Gregers Hasselbalch, Anne Byriel Walls, Anja Hviid Simonsen

**Affiliations:** ^1^ Danish Dementia Research Centre, Department of Neurology Copenhagen University Hospital, Rigshospitalet Copenhagen Denmark; ^2^ Regional Dementia Research Centre, Department of Neurology Zealand University Hospital Roskilde Denmark; ^3^ Department of Clinical Medicine University of Copenhagen Copenhagen Denmark; ^4^ Department of Psychiatry and Neurochemistry, Institute of Neuroscience and Physiology Sahlgrenska Academy at the University of Gothenburg Mölndal Sweden; ^5^ Institute of Psychiatry, Psychology and Neuroscience Maurice Wohl Institute Clinical Neuroscience Institute King's College London London UK; ^6^ NIHR Biomedical Research Centre for Mental Health and Biomedical Research Unit for Dementia at South London and Maudsley NHS Foundation London UK; ^7^ Centre for Age‐Related Medicine Stavanger University Hospital Stavanger Norway; ^8^ Clinical Neurochemistry Laboratory Sahlgrenska University Hospital Mölndal Sweden; ^9^ Department of Neurodegenerative Disease UCL Institute of Neurology, Queen Square London UK; ^10^ UK Dementia Research Institute at UCL London UK; ^11^ Hong Kong Center for Neurodegenerative Diseases Clear Water Bay Hong Kong China; ^12^ Wisconsin Alzheimer's Disease Research Center, University of Wisconsin School of Medicine and Public Health University of Wisconsin–Madison Madison Wisconsin USA; ^13^ Paris Brain Institute, ICM, Pitié‐Salpêtrière Hospital Sorbonne University Paris France; ^14^ Neurodegenerative Disorder Research Center, Division of Life Sciences and Medicine, and Department of Neurology, Institute on Aging and Brain Disorders University of Science and Technology of China and First Affiliated Hospital of USTC Hefei China; ^15^ Department of Drug Design and Pharmacology, Faculty of Health and Medical Sciences University of Copenhagen Copenhagen Denmark; ^16^ Capital Region Hospital Pharmacy Rigshospitalet Copenhagen Denmark

**Keywords:** Alzheimer's disease, CSF, diagnosis, SNAP‐25

## Abstract

Synaptic degeneration has been linked to cognitive decline. The presynaptic protein, synaptosomal‐associated protein 25 kDA (SNAP‐25), is crucial for synaptic transmission and has been suggested as a biomarker in Alzheimer's disease (AD). In the current study, we investigated the ability of SNAP‐25 to differentiate between heterogenous dementia etiologies and whether SNAP‐25 could be a staging marker in AD. SNAP‐25 in the cerebrospinal fluid (CSF) from a retrospective (*n* = 187) and a prospective (*n* = 134) cohort was investigated with immunoprecipitation mass spectrometry (IP‐MS) and single‐molecule array (Simoa), respectively. Both cohorts consisted of healthy controls (HC) and patients with cognitive decline of different etiologies. CSF SNAP‐25 concentration was higher in AD and non‐neurodegenerative diseases (i.e., vascular dementia) compared with controls but did not differ between AD and non‐AD neurodegenerative diseases. We found a trend toward an association between SNAP‐25 and disease burden when comparing HC, mild cognitive impairment due to AD, and AD. CSF SNAP‐25 concentrations were strongly associated with CSF phosphorylated tau (p‐tau) concentrations, thus strengthening the link between synaptic dysfunction and tau pathophysiology in AD. Our initial findings suggest that SNAP‐25 may be a potential biomarker for differentiating AD from dementia due to other etiologies. However, due to the significant association between SNAP‐25 and p‐tau proteins, the clinical utility of SNAP‐25 as a diagnostic biomarker for AD may be limited, while SNAP‐25 may be useful for monitoring disease progression or treatment response.

## Graphical abstract

Cerebrospinal Fluid levels of Synaptosomal Associated Protein 25 kDA (SNAP‐25) were elevated in AD and non‐neurodegenerative diseases compared to controls but did not distinguish AD from other neurodegenerative diseases. SNAP‐25 correlated strongly with CSF phosphorylated tau, indicating a link between synaptic dysfunction and tau pathology. Although SNAP‐25 has limited diagnostic value for AD, it may be useful for monitoring disease progression or treatment response.

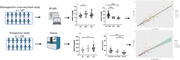

## 1 Introduction

In patients with Alzheimer's disease (AD), synaptic degeneration has been linked to cognitive decline (Camporesi et al. 2020). Specifically, due to the relationship between synaptic degeneration and cognitive changes, synaptic proteins have been suggested as biomarkers for disease staging and prediction of progression (Davidsson, Puchades, and Blennow 1999; Terry et al. 1991). The exocytotic release of neurotransmitters during synaptic transmission is controlled by a process including the soluble *N*‐ethylmaleimide‐sensitive factor attachment protein receptor (SNARE) protein complex (Antonucci et al. 2016; Margiotta 2021). Synaptosomal‐associated protein 25 kDa (SNAP‐25), synaptobrevin, and syntaxin‐1 are part of the SNARE protein complex in the presynaptic membrane, which promotes synaptic communication by initiating the fusion of synaptic vesicles to the postsynaptic membrane (Margiotta 2021). SNAP‐25 is widely distributed in the synapses throughout the central nervous system (CNS) and has several functions, including synaptogenesis and repair of neurons. A loss of SNAP‐25 might not halt the neurotransmission per se but could instead cause physiological changes in synaptic transmission, leading to synaptic damage (Bark et al. 1995). Other studies have shown that SNAP‐25 also influences postsynaptic functions such as spine morphogenesis and plasticity (Antonucci et al. 2016).

Due to these dual pre‐ and postsynaptic roles, SNAP‐25 has been suggested as a biomarker for neurodegeneration.

To understand the role of SNAP‐25 as a biomarker for AD, it is essential to understand its association with conventional AD biomarkers. Previous studies suggest a significant association between tau and SNAP‐25 (Brinkmalm et al. 2014; Morar et al. 2022; Öhrfelt et al. 2019; Tible et al. 2020).

One study suggested that SNAP‐25 in cerebrospinal fluid (CSF) represents synaptic degeneration, which is affected by amyloid and tau (Benedet et al. 2020), while other studies suggest that the loss of synapses precedes the loss of neurons, which therefore alters the levels of SNAP‐25 in the preclinical stages (Zhang et al. 2018). More recently, a meta‐analysis has shown that SNAP‐25 levels in CSF differentiated patients with MCI or AD from healthy controls (HC). Furthermore, correlations were found when comparing CSF total tau (t‐tau) and phosphorylated tau (p‐tau) with CSF SNAP‐25 (Liu et al. 2022), highlighting the strong link between tau pathophysiology and synaptic loss.

To understand the role and validity of SNAP‐25 in differentiating between dementias of different etiologies, two separate cohorts were included in this study. These were a retrospective cohort with selected samples from the Danish Dementia Biobank and a consecutive cohort with prospectively collected samples to validate the findings of the retrospective cohort in a real clinical scenario. The main objectives were, first, to investigate whether CSF SNAP‐25 can differentiate between disease stages in AD and between cognitive decline of different etiologies and, second, to understand the clinical applicability of SNAP‐25 by investigating the association between SNAP‐25 and conventional AD biomarkers.

## 2 Methods

### 2.1 Overview of Cohort Studies

#### 2.1.1 Retrospective Cross‐Sectional Study

This study included a selected group of 187 patients, who had donated a CSF sample between March 2008 and April 2016. The participants were selected if they had available CSF fluid in the biobank as well as data from routine clinical biochemistry. The patients gave informed consent for their data and biological samples to be used for research purposes during the diagnostic evaluation at the Copenhagen Memory Clinic, Copenhagen University Hospital, Rigshospitalet.

CSF samples from HC (*n* = 38) and patients with mild cognitive impairment due to prodromal AD (MCI_AD) (*n* = 35), dementia due to AD (*n* = 56), vascular dementia (VaD) (*n* = 29), and normal pressure hydrocephalus (NPH) (*n* = 29) were selected for SNAP‐25 analysis. Patients were diagnosed after clinical assessments, neuropsychological examination, and structural imaging (magnetic resonance imaging [MRI] or computed tomography [CT]), supplemented with ^18^F‐fluorodeoxyglucose positron emission tomography [^18^F‐FDG‐PET]) when indicated. HC were referred to the memory clinic as patients but were found to be cognitively normal and to have no evidence of neurodegenerative pathology.

Patients diagnosed with AD between 2008 and 2012 fulfilled the National Institute of Neurological and Communicative Disorders and Stroke and the Alzheimer's Disease and Related Disorders Association (NINCDS‐ADRDA) criteria (G. McKhann et al. 1984), and from 2013 to 2016, they fulfilled the National Institute of Aging and the Alzheimer's Association (NIA‐AA) criteria (G. M. McKhann et al. 2011). Patients diagnosed with MCI between 2008 and 2012 fulfilled Petersen 2004 and the NINCDS‐ADRDA criteria (G. McKhann et al. 1984; Petersen 2004), and from 2013 to 2016, they fulfilled the NIA‐AA criteria for MCI due to AD (Albert et al. 2011). Patients diagnosed with VaD between 2008 and 2013 fulfilled the National Institute of Neurological Disorders and Stroke and the Association Internationale pour la Recherche et l'Enseignement en Neurosciences (NINDS‐AIREN) criteria (Van Straaten et al. 2003), and from 2014 to 2016, they fulfilled the International Society for Vascular Behavioral and Cognitive Disorders (VASCOG) criteria (Sachdev et al. 2014). Patients with NPH met the international guideline criteria for iNPH (Relkin et al. 2005). All included participants underwent a lumbar puncture as part of their diagnostic assessment and were classified according to local threshold values (Simonsen et al. 2021).

#### 2.1.2 Prospective Study

This study included a total of 134 patients recruited between March and December 2019. The patients were recruited either from the Copenhagen Memory Clinic, Copenhagen University Hospital, Rigshospitalet or from the Regional Dementia Research Center, Zealand University Hospital, Roskilde.

CSF samples were collected by lumbar puncture from HC (*n* = 14), MCI_AD (*n* = 10), AD (*n* = 50), non‐AD neurodegenerative diseases (*n* = 18), and non‐neurodegenerative diseases (*n* = 42). The group of non‐AD neurodegenerative diseases consists of individuals with dementia with Lewy Bodies (DLB) (*n* = 5), frontotemporal dementia (FTD) (*n* = 6), MCI with DLB etiology (*n* = 3), MCI with FTD etiology (*n* = 1), progressive supranuclear palsy (PSP) (*n* = 1), MCI with PSP (*n* = 1), and primary progressive multiple sclerosis (PPMS) (*n* = 1). The group of non‐neurodegenerative diseases consists of individuals with VaD (*n* = 7), NPH (*n* = 10), MCI with VaD etiology (*n* = 5), MCI with NPH etiology (*n* = 9), alcohol dementia (*n* = 3), MCI due to alcohol dementia (*n* = 1), MCI with non‐neurodegenerative disease (*n* = 6), and other non‐neurodegenerative disease (*n* = 1). All patients were diagnosed after clinical and neuropsychological examination and imaging (MRI, CT, and, in most subjects, ^18^F‐FDG‐PET). CSF Ab42, t‐tau, and p‐tau biomarkers were used for diagnostic purposes. Patients diagnosed with AD, MCI due to prodromal AD, VaD, and NPH were diagnosed as described above. In addition, the patients with MCI fulfilled the criteria of the International Working Group on Mild Cognitive Impairment (Winblad et al. 2004). DLB was diagnosed according to the fourth report of the DLB consortium, (Mckeith et al. 2017), while the criteria for the behavioral variant, the non‐fluent aphasia, or the semantic variant were fulfilled for patients diagnosed with FTD (Gorno‐Tempini et al. 2011; Rascovsky et al. 2011). The criteria of the International Parkinson and Movement Disorder Society were fulfilled for patients diagnosed with PSP (Höglinger et al. 2017), and patients diagnosed with alcoholic dementia were scored according to the International Statistical Classification of Diseases and Related Health Problems 10^th^ Revision (ICD‐10) (WHO 2010). The HC consisted of patients, who were either found to be cognitively healthy with no clinical or pathological signs of a neurological disease or subjects recruited for research purposes only. All HC were Aβ− and did not meet the criteria for MCI or dementia.

The study was designed in accordance with the declaration of Helsinki, and all patients gave written consent for their samples and clinical data to be used for research. This study was approved by the Ethical Committee of the Capital Region of Denmark (H‐16041133, H‐19000651) and the Danish Data Protection Agency (RH‐2016‐371, VD‐2019‐105).

### 2.2 CSF Sampling and Biomarker Assays for Both Cohorts

All SNAP‐25 analyses were performed at the Department of Psychiatry and Neurochemistry, Sahlgrenska Academy, University of Gothenburg. For both cohorts, CSF samples were obtained through lumbar puncture and were collected in polypropylene tubes (Del Campo et al. 2012; Engelborghs et al. 2017). The samples were centrifuged at 2000 rpm for 10 min at 4°C. Thereafter, they were redistributed in 250‐µL aliquots and stored at −80°C. CSF Aβ42, t‐tau, and p‐tau were determined using sandwich enzyme‐linked immunosorbent assay (ELISA) INNOTEST b‐AMYLOID (1–42) Fujirebio, INNOTEST hTau Ag, and INNOTEST PHOSPHO‐TAU (181P), respectively. In the retrospective cross‐sectional study, SNAP‐25 levels were measured using immunoprecipitation mass spectrometry (IP‐MS), while the prospective study used single‐molecule array (Simoa) (Nilsson et al. 2022).

### 2.3 Statistical Analyses

The statistical analyses were performed in R (v4.1.0). A one‐way analysis of variance (ANOVA) was used to compare age, Mini‐Mental State Examination (MMSE) score, t‐tau, p‐tau, and SNAP‐25 between the groups. If the ANOVA test was significant between groups, post hoc *t*‐tests were computed based on the model (reported in the Supporting Information). To compare SNAP‐25 levels, we performed an analysis of covariance (ANCOVA) with age and sex as covariates if they were significantly different between groups. The SNAP‐25 values were log‐transformed due to the nonnormal distribution of the residuals. If significant, a post hoc Tukey's test was performed using the *multicompare* package in R. The association between SNAP‐25 and either t‐tau or p‐tau was investigated using separate linear regression models. Statistical tests were considered significant with a *p*‐value < 0.05. Code and output from R can be found in the Supporting Information.

## 3 Results

### 3.1 Patient Characteristics

Patient characteristics can be found in Table 1 for the retrospective cohort (*n* = 187) and in Table 2 for the prospective cohort (*n* = 134).

**TABLE 1:** Patient characteristics for the retrospective cross‐sectional study.

Characteristics	HC (*n* = 38)	MCI (*n* = 35)	AD (*n* = 56)	VaD (*n* = 29)	NPH (*n* = 29)	*p*‐value
Sex distribution; F/M	16/22	15/20	26/30	6/23	10/19	0.198^b^
						0.902^c^
						0.057^d^
Aβ42 status; Aβ−/Aβ+	28/3^a^	10/24^a^	17/39^a^	14/14^a^	8/15^a^	< 0.01^b^
Age [years]; mean (SD)	65.1 (8.3)	69.7 (7.9)	70.1 (8.3)	71.6 (7.7)	72.2 (6.0)	< 0.01^b^
						< 0.05^c^
						0.314^d^
MMSE; mean (SD)	29.1 (1.3)	26.8 (1.9)	21.8 (5.3)	24.6 (3.5)	26.9 (3.1)	< 0.001^b^
t‐tau [pg/mL]; mean (SD)	339.0 (205.4)	485.1 (236.1)	436.4 (244.6)	267.1 (151.9)	222.4 (216.7)	< 0.001^b^
p‐tau [pg/mL]; mean (SD)	50.1 (28.4)	74.7 (29.4)	69.3 (28.6)	35.3 (15.6)	28.2 (11.7)	< 0.001^b^
SNAP‐25 [pM]; mean (SD)	13.0 (2.4)	14.8 (2.8)	14.4 (3.2)	10.7 (1.8)	10.0 (1.3)	< 0.001^b^, ^e^

Abbreviations: Aβ42, amyloid beta 1–42; AD, Alzheimer's disease; HC, healthy controls; MCI, mild cognitive impairment due to prodromal AD; MMSE, Mini‐Mental State Examination; VaD: Vascular dementia, NPH: Normal pressure hydrocephalus; p‐tau, phosphorylated tau; SNAP‐25, synaptosomal‐associated‐protein 25 kDA; t‐tau, total‐tau.

^a^ Data missing from seven out of 38 HC, one out of 35 MCI, one out of 29 VaD, and six out of 29 NPH.

^b^ The *p*‐value for the whole cohort.

^c^ The *p*‐value for the analysis of HC, MCI, and AD.

^d^ The *p*‐value for the analysis of AD/MCI, VaD, and NPH.

^e^ The *p*‐value with age as covariate.

**TABLE 2:** Patient characteristics for the prospective study.

Characteristics	HC (*n* = 14)	MCI (*n* = 10)	AD (*n* = 50)	Non‐AD‐ND (*n* = 18)	Non‐ND (*n* = 42)	*p*‐value
Sex distribution; F/M	5/9	8/2	31/19	7/11	10/32	< 0.001^b^
						0.076^c^
						< 0.001^d^
Aβ42 status; Aβ−/Aβ+	4/2^a^	1/9	3/47	11/7	20/22	< 0.001^b^
Age [years]; mean (SD)	67.5 (8.7)	68.5 (9.7)	72.4 (7.8)	69.9 (7.9)	74.0 (7.3)	< 0.05^b^
						0.090^c^
						0.145^d^
MMSE; mean (SD)	28.0 (1.7)	26.8 (2.8)	22.8 (4.2)	24.9 (5.0)	24.0 (4.5)	< 0.01^b^
t‐tau [pg/mL]; mean (SD)	293.8 (131.1)	523.7 (241.9)	603.5 (277.1)	346.4 (149.9)	260.1 (178.3)	< 0.001^b^
p‐tau [pg/mL]; mean (SD)	54.8 (24.2)	73.2 (23.8)	88.6 (45.9)	53.5 (26.2)	46.6 (23.9)	< 0.001^b^
SNAP‐25 [pg/mL]; mean (SD)	130.8 (44.4)	173.4 (64.8)	195.4 (74.5)	154.6 (50.5)	111.2 (83.2)	< 0.001^b^, ^e^

Abbreviations: Aβ42, amyloid beta 1–42; AD, Alzheimer's disease; HC, healthy controls; MCI, mild cognitive impairment due to prodromal AD; MMSE, Mini‐Mental State Examination; Non‐AD‐ND, non‐Alzheimer's disease neurodegenerative diseases; Non‐ND, non‐neurodegenerative diseases dementia; p‐tau, phosphorylated tau; SNAP‐25, synaptosomal‐associated‐protein 25 kDA; t‐tau, total‐tau.

^a^ Data missing from eight out of 14 HC.

^b^ The *p*‐value for the whole cohort.

^c^ The *p*‐value for the analysis of AD disease staging (HC, MCI, and AD).

^d^ The *p*‐value for the comparison between AD, non‐AD ND disease, and non‐ND dementia.

^e^ The *p*‐value with age and sex as covariates.

### 3.2 CSF SNAP‐25 Levels in HC, MCI, and AD

**FIGURE 1:**
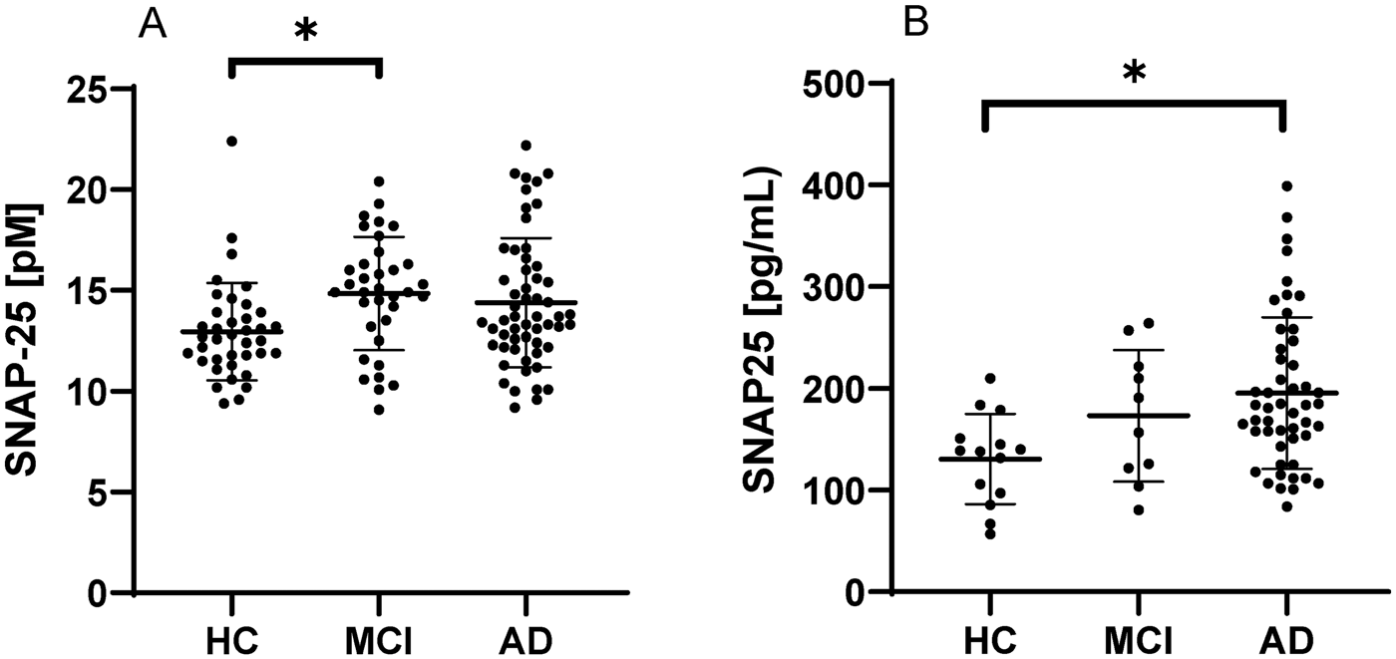
(A) Retrospective cohort. CSF SNAP‐25 in the different diagnostic groups: HC (*n* = 38), MCI due to prodromal AD (*n* = 35), and AD (*n* = 56). (B) Prospective cohort. Scatterplot of CSF SNAP‐25 in the different diagnostic groups: HC (*n* = 14), MCI due to prodromal AD (*n* = 10), and AD (*n* = 50). The plots show the mean and standard deviation for each of the three groups. *p < 0.05.

In the retrospective cohort, CSF SNAP‐25 levels were significantly different between disease stages (AD, MCI due to prodromal AD, and HC) (*p* < 0.05, *F*‐value = 3.55, df = 2) with age as a covariate. Post hoc comparisons showed that SNAP‐25 was significantly higher in MCI versus HC (95% CI: 0.01–0.24, *p* < 0.05). No significant differences were observed in CSF SNAP‐25 levels in HC versus AD (95% CI: −0.19 to 0.02, *p* = 0.118) and MCI versus AD (95% CI: −0.06 to 0.14, *p* = 0.667) (see Figure 1A).

In the prospective study, CSF SNAP‐25 levels were significantly different between the groups (*p* < 0.05, *F*‐value = 6.03, df = 2). Post hoc comparisons showed that CSF SNAP‐25 levels were significantly different in HC versus AD (95% CI: −0.67 to −0.12, *p* < 0.05). There was no significant difference in MCI due to prodromal AD versus AD (95% CI: −0.43 to 0.19, *p* = 0.621) and in MCI versus HC (95% CI: −0.10 to 0.65, *p* = 0.188) (see Figure 1B).

### 3.3 Comparison of SNAP‐25 Between AD and Dementia due to Other Etiologies

**FIGURE 2:**
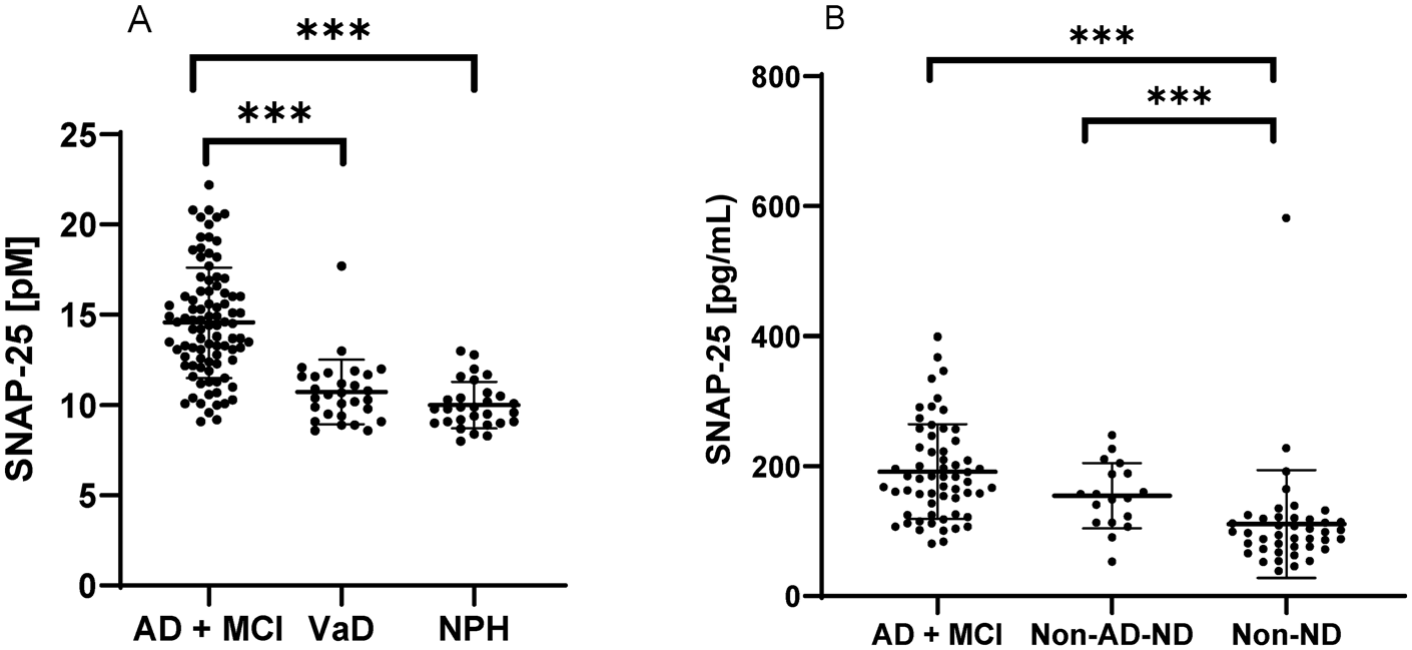
(A) CSF SNAP‐25 in AD+MCI due to prodromal AD group (*n* = 91), VaD (*n* = 29), and NPH (*n* = 29). (B) CSF SNAP‐25 in the different diagnostic groups: AD+MCI due to prodromal AD (*n* = 60), non‐AD‐ND (*n* = 18), and non‐ND (*n* = 42). Depicted mean and standard deviation. AD, Alzheimer's disease; MCI, mild cognitive impairment due to prodromal Alzheimer's disease; VaD, vascular dementia; NPH, normal pressure hydrocephalus; Non‐AD‐ND, non‐Alzheimer's disease neurodegenerative diseases; Non‐ND, non‐neurodegenerative diseases; SNAP‐25, synaptosomal‐associated protein 25 kDA. ***p < 0.001.

In the retrospective cohort, we found that CSF SNAP‐25 levels were significantly different between AD and MCI due to prodromal AD, VaD, and NPH (*p* < 0.001, *F*‐value = 56.39, df = 2). Post hoc comparisons showed that CSF SNAP‐25 levels were significantly higher in VaD versus AD combined with MCI due to prodromal AD (95% CI: −0.39 to −0.20, *p* < 0.001) and in NPH versus AD combined with MCI due to prodromal AD (95% CI: −0.45 to −0.27, *p* < 0.001). No significant differences were observed in CSF SNAP‐25 levels in NPH versus VaD (95% CI: −0.18 to 0.05, *p* = 0.356) (see Figure 2A).

In the prospective cohort, the SNAP‐25 levels between AD and MCI due to prodromal AD (*n* = 60), non‐AD‐ND (*n* = 18), and non‐ND (*n* = 42) were significantly different (*p* < 0.001, *F*‐value = 22.70, df = 2). Post hoc comparisons showed that CSF SNAP‐25 levels were significantly higher in non‐ND versus AD combined with MCI due to prodromal AD (95% CI: −0.81 to −0.39, *p* < 0.001) and in non‐ND versus non‐AD‐ND (95% CI: −0.67 to −0.12, *p* < 0.05). There was no statistical significance between non‐AD‐ND versus AD combined with MCI due to prodromal AD (95% CI: −0.46 to 0.07, *p* = 0.182) (see Figure 2B).

### 3.4 Associations of CSF SNAP‐25 With p‐tau and t‐tau

**FIGURE 3:**
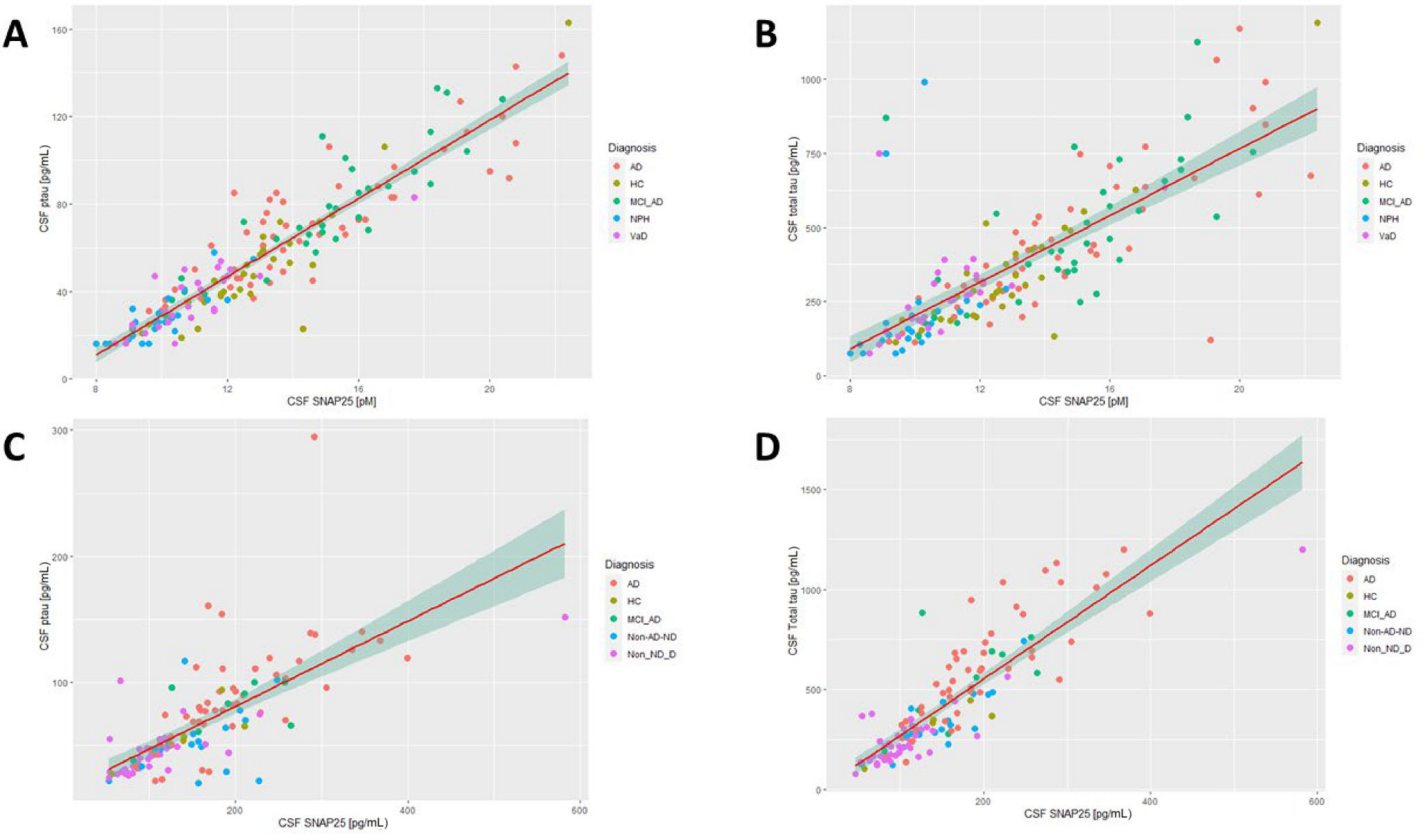
CSF SNAP‐25 levels in relation to p‐tau and t‐tau in the retrospective cohort (A, B) and prospective cohort (C, D). HC, healthy controls; MCI_AD, mild cognitive impairment due to prodromal Alzheimer's disease; AD, Alzheimer's disease; VaD, vascular dementia; NPH, normal pressure hydrocephalus; Non‐AD‐ND, non‐Alzheimer's disease neurodegenerative diseases; Non‐ND‐D, non‐neurodegenerative diseases; SNAP‐25, synaptosomal‐associated protein 25 kDA.

Using linear regression in both cohorts, we found that, in the retrospective cohort, CSF SNAP‐25 was significantly associated with p‐tau (estimate = 0.095, *p* < 0.001) and t‐tau (estimate = 0.010, *p* < 0.001). In the prospective cohort, a similar result was found. Here, CSF SNAP‐25 was significantly associated with p‐tau (estimate = 1.443, *p* < 0.001) and t‐tau (estimate = 0.255, *p* < 0.001) (see Figure 3A–D).

## 4 Discussion

The objective of the present study was to investigate if CSF SNAP‐25 could differentiate between AD disease stages and between cognitive decline of different etiologies. Furthermore, to understand the utility of this biomarker, we investigated the association between SNAP‐25 and conventional AD biomarkers.

The potential utility of SNAP‐25 as a biomarker for AD staging remains inconclusive based on our findings using a retrospective cohort of selected samples and a prospective consecutive cohort, which more closely reflects the real clinical setting. In the retrospective study, significantly elevated SNAP‐25 levels were seen in MCI due to prodromal AD compared to both HC and AD, although less cognitive decline was observed in MCI due to prodromal AD as compared to AD, as indicated by the MMSE score, as expected. In contrast, the prospective study showed a significant increase in SNAP‐25 in AD compared to both MCI due to prodromal AD and HC. These contradictory results warrant further investigation before considering the value of SNAP‐25 as a marker for AD staging. A recent systematic review of the literature concluded that patients with AD showed elevated levels of SNAP‐25 compared to HC, but there was substantial heterogeneity between the four studies available for review (Roveta et al. 2022).

We investigated the discriminatory capacity of SNAP‐25 in distinguishing between cognitive decline of different etiologies. Our analysis of two clinical cohorts showed that SNAP‐25 could differentiate between patients with AD and the non‐neurodegenerative diseases NPH and VaD but lacked the ability to differentiate between the AD and non‐AD neurodegenerative diseases.

A study by Tible et al. reported lower SNAP‐25 levels in patients with dementias other than AD, although their non‐AD group contained both neurodegenerative and non‐neurodegenerative diseases (Tible et al. 2020). Further, Nilsson et al. assessed the ability of SNAP‐25 to detect amyloid positivity in a cohort of cognitively impaired and unimpaired individuals, yielding an area under the receiver operator curve (AUC) of 0.74 (Nilsson et al. 2022). Combined with the current findings, it seems likely that SNAP‐25 levels in CSF are affected in other neurodegenerative disorders, including disorders not involving AD pathology.

In the present study, we also observed a strong association between SNAP‐25 and tau proteins. Our results from both the retrospective cross‐sectional study and the prospective study showed a positive association between SNAP‐25 and both t‐tau and p‐tau. The association could suggest that synaptic dysfunction and tau pathology are strongly linked in AD (Wu et al. 2021). Moreover, SNAP‐25 could play a role as a marker of subcortical denervation. However, this study cannot reasonably investigate this. Based on these findings, it is unlikely that SNAP‐25 will be useful as a diagnostic marker for AD in a clinical setting. In contrast, the prognostic value of SNAP‐25 for disease monitoring remains to be investigated.

The strengths of this study were the employment of two independent sample cohorts: a retrospective cross‐sectional study and a prospective study with consecutive inclusion, both including well‐characterized individuals with typical clinical presentations of memory decline in a real‐world setting. However, it is relevant to acknowledge certain limitations associated with these clinical cohorts, namely their heterogeneity and relatively limited data in comparison with research cohorts. This may explain some of the findings, including a higher level of SNAP‐25 in the MCI due to prodromal AD group in the retrospective cohort.

In conclusion, our results show that SNAP‐25 may be a potential biomarker for differentiating AD from non‐neurodegenerative diseases. However, due to the significant association between SNAP‐25 and tau proteins, the role of SNAP‐25 in AD diagnosis is limited. Future studies are needed to ascertain whether SNAP‐25 could contribute to the diagnostic differentiation in other neurodegenerative diseases or be used for monitoring the response to disease‐modifying treatments for AD.

## Author Contributions

**Sofia Hjorth Wolner**: writing–original draft, formal analysis. **Helena Sophia Gleerup**: validation, formal analysis, writing–review and editing. **Christian Sandøe Musaeus**: data curation, validation, formal analysis, writing–review and editing. **Peter Høgh**: resources, writing–review and editing. **Nicholas J. Ashton**: resources, methodology, writing–review and editing. **Ann Brinkmalm**: methodology, investigation, writing–review and editing. **Johanna Nilsson**: methodology, investigation, writing–review and editing. **Lana Grötschel**: methodology, investigation, writing–review and editing. **Henrik Zetterberg**: resources, methodology, funding acquisition, writing–review and editing. **Kaj Blennow**: resources, methodology, funding acquisition, writing–review and editing. **Steen Gregers Hasselbalch**: resources, funding acquisition, writing–review and editing. **Anne Byriel Walls**: writing–review and editing. **Anja Hviid Simonsen**: conceptualization, supervision, writing–review and editing.

## Conflicts of Interest

H.Z. has served at scientific advisory boards and/or as a consultant for Abbvie, Acumen, Alector, Alzinova, ALZPath, Amylyx, Annexon, Apellis, Artery Therapeutics, AZTherapies, Cognito Therapeutics, CogRx, Denali, Eisai, Merry Life, Nervgen, Novo Nordisk, Optoceutics, Passage Bio, Pinteon Therapeutics, Prothena, Red Abbey Labs, reMYND, Roche, Samumed, Siemens Healthineers, Triplet Therapeutics, and Wave; has given lectures in symposia sponsored by Alzecure, Biogen, Cellectricon, Fujirebio, Lilly, Novo Nordisk, and Roche; and is a co‐founder of Brain Biomarker Solutions in Gothenburg AB (BBS), which is a part of the GU Ventures Incubator Program (outside submitted work). K.B. has served as a consultant and at advisory boards for Abbvie, AC Immune, ALZPath, AriBio, BioArctic, Biogen, Eisai, Lilly, Moleac Pte. Ltd, Neurimmune, Novartis, Ono Pharma, Prothena, Roche Diagnostics, and Siemens Healthineers; has served at data monitoring committees for Julius Clinical and Novartis; has given lectures, produced educational materials, and participated in educational programs for AC Immune, Biogen, Celdara Medical, Eisai, and Roche Diagnostics; and is a co‐founder of Brain Biomarker Solutions in Gothenburg AB (BBS), which is a part of the GU Ventures Incubator Program, outside the work presented in this paper. The other authors declare no conflicts of interest.

## Supporting information



Supplementary Materials.

## Data Availability

The data that support the findings of this study are available on request from the corresponding author. The data are not publicly available due to privacy or ethical restrictions.
