# Supplementary material for: Synaptosomal‐Associated Protein 25 kDA (SNAP‐25) Levels in Cerebrospinal Fluid: Implications for Alzheimer's Disease Diagnosis and Monitoring
Source: Synapse. 2025 Feb 6;79(2):e70010. doi: 10.1002/syn.70010 (PMC11800177; doi:10.1002/syn.70010)
Supplement: Supplementary file 1 — Supplementary Materials. [file SYN-79-e70010-s001.docx]

SNAP-25

See author list

2024-01-25

# Retrospective

library(car)

library(readxl)

library(dplyr)

library(tidyverse)

#Loading of Excel file
setwd("P:/RH/NEU/Lukkede Mapper/BiobankCSFProjekt2017/SNAP-25/Statistik_mappe/R/Tværsnit")
SNAP25 <- read_excel('old cohort_with values.xlsx')

# chi squared test for sex distribution between the groups

table1 <- table(SNAP25$Sex, SNAP25$Diagnosis)
table1

##
## AD HC MCI_AD NPH VaD
## F 26 16 15 10 6
## M 30 22 20 19 23

chisq.test(table1)

##
## Pearson's Chi-squared test
##
## data: table1
## X-squared = 6.0054, df = 4, p-value = 0.1987

# chi squared test for amyloid status between groups

table2 <- table(SNAP25$AB42_Status, SNAP25$Diagnosis)
table2

##
## AD HC MCI_AD NPH VaD
## AB_negative 17 28 10 8 14
## AB_positive 39 3 24 15 14

chisq.test(table2)

##
## Pearson's Chi-squared test
##
## data: table2
## X-squared = 35.201, df = 4, p-value = 4.224e-07

# one way ANOVA for age between groups - with post-hoc pairwise t-test:

SNAP25$Diagnosis <- as.factor(SNAP25$Diagnosis)

model_age <- aov(AgeAtVisit~Diagnosis,data = SNAP25)

plot(model_age)


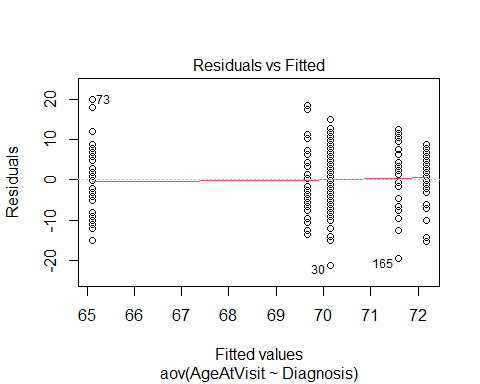

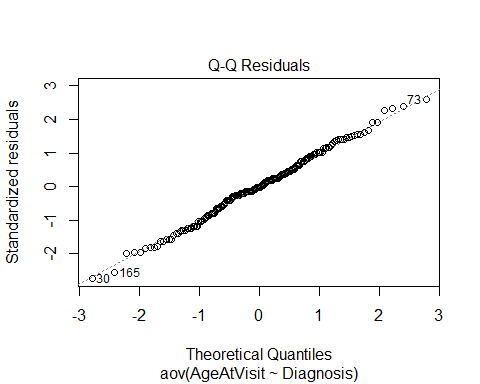

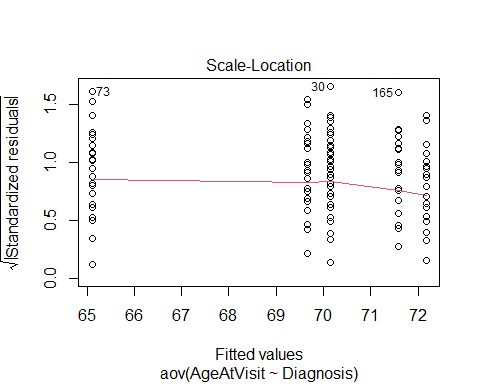

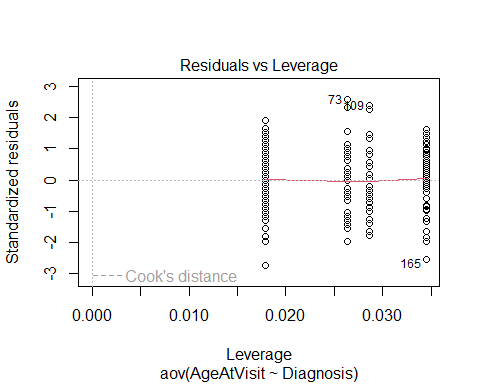


summary(model_age)

## Df Sum Sq Mean Sq F value Pr(>F)
## Diagnosis 4 1090 272.61 4.484 0.00177 **
## Residuals 182 11063 60.79
## ---
## Signif. codes: 0 '***' 0.001 '**' 0.01 '*' 0.05 '.' 0.1 ' ' 1

Anova(model_age, type = "II")

## Anova Table (Type II tests)
##
## Response: AgeAtVisit
## Sum Sq Df F value Pr(>F)
## Diagnosis 1090.4 4 4.4845 0.001767 **
## Residuals 11063.5 182
## ---
## Signif. codes: 0 '***' 0.001 '**' 0.01 '*' 0.05 '.' 0.1 ' ' 1

attach(SNAP25)

pairwise.t.test(AgeAtVisit, Diagnosis, p.adj = "none")

##
## Pairwise comparisons using t tests with pooled SD
##
## data: AgeAtVisit and Diagnosis
##
## AD HC MCI_AD NPH
## HC 0.00244 - - -
## MCI_AD 0.77282 0.01360 - -
## NPH 0.25669 0.00031 0.20051 -
## VaD 0.41947 0.00091 0.32577 0.77497
##
## P value adjustment method: none

tapply(AgeAtVisit, Diagnosis, mean, na.rm=TRUE)

## AD HC MCI_AD NPH VaD
## 70.14286 65.10526 69.65714 72.17241 71.58621

tapply(AgeAtVisit, Diagnosis, sd, na.rm=TRUE)

## AD HC MCI_AD NPH VaD
## 8.253767 8.343275 7.870058 5.964592 7.650944

# one way ANOVA for MMSE between groups - with post-hoc pairwise t-test:

SNAP25$Diagnosis <- as.factor(SNAP25$Diagnosis)

model_mmse <- aov(MMSE~Diagnosis,data = SNAP25)

plot(model_mmse)


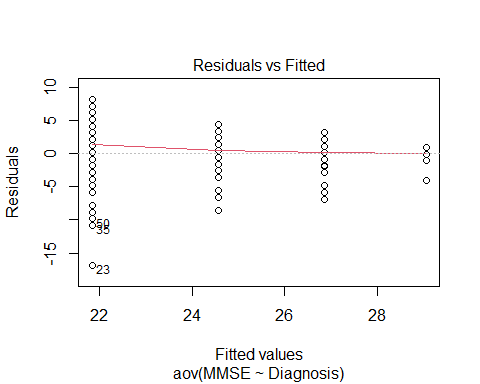

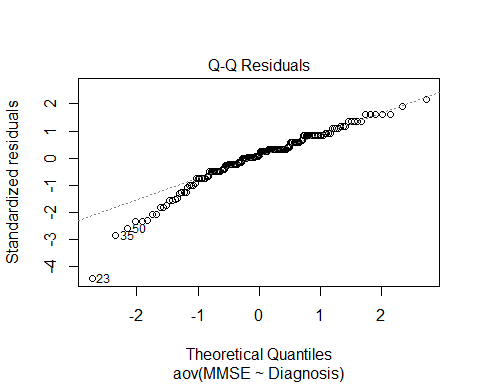

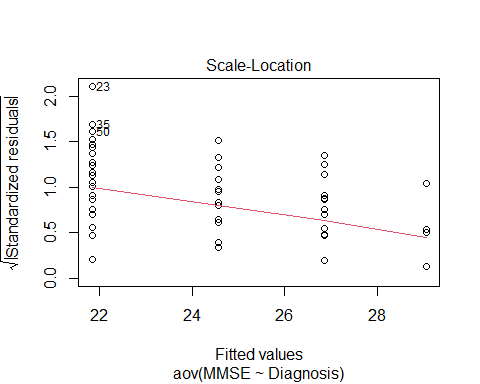

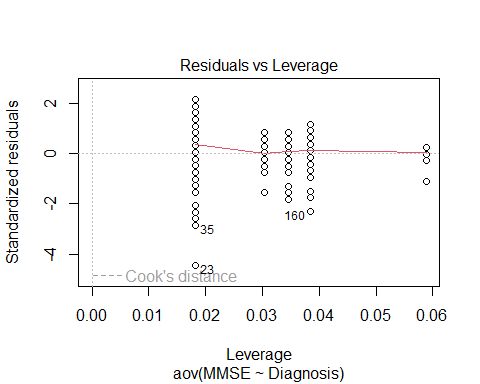


summary(model_mmse)

## Df Sum Sq Mean Sq F value Pr(>F)
## Diagnosis 4 1048 262.12 17.91 4.16e-12 ***
## Residuals 155 2268 14.64
## ---
## Signif. codes: 0 '***' 0.001 '**' 0.01 '*' 0.05 '.' 0.1 ' ' 1
## 27 observations deleted due to missingness

Anova(model_mmse, type = "II")

## Anova Table (Type II tests)
##
## Response: MMSE
## Sum Sq Df F value Pr(>F)
## Diagnosis 1048.5 4 17.91 4.155e-12 ***
## Residuals 2268.5 155
## ---
## Signif. codes: 0 '***' 0.001 '**' 0.01 '*' 0.05 '.' 0.1 ' ' 1

attach(SNAP25)

## De følgende objekter er maskerede fra SNAP25 (pos = 3):
##
## AB42_Status, AgeAtVisit, CognitiveSyndrome, CSF_ABeta42,
## CSF_PhosphoTau, CSF_SNAP25, CSF_TotalTau, DDBBno, Diagnosis, Group,
## MMSE, Sex, VisitDate

pairwise.t.test(MMSE, Diagnosis, p.adj = "none")

##
## Pairwise comparisons using t tests with pooled SD
##
## data: MMSE and Diagnosis
##
## AD HC MCI_AD NPH
## HC 2.1e-10 - - -
## MCI_AD 1.7e-08 0.05477 - -
## NPH 5.2e-08 0.06201 0.98889 -
## VaD 0.00305 0.00024 0.02494 0.02846
##
## P value adjustment method: none

tapply(MMSE, Diagnosis, mean, na.rm=TRUE)

## AD HC MCI_AD NPH VaD
## 21.83636 29.05882 26.84848 26.86207 24.57692

tapply(MMSE, Diagnosis, sd, na.rm=TRUE)

## AD HC MCI_AD NPH VaD
## 5.339454 1.297622 1.938447 3.136469 3.500549

# one way ANOVA for t-tau between groups - with post-hoc pairwise t-test:

SNAP25$Diagnosis <- as.factor(SNAP25$Diagnosis)

model_ttau <- aov(CSF_TotalTau~Diagnosis,data = SNAP25)

plot(model_ttau)


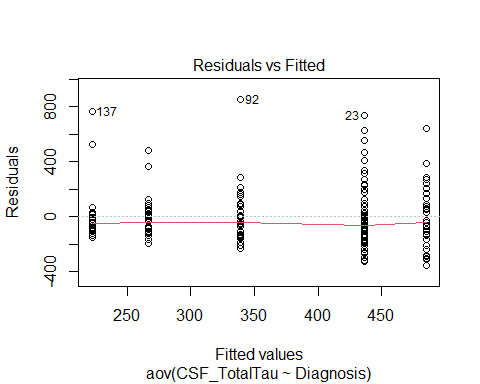

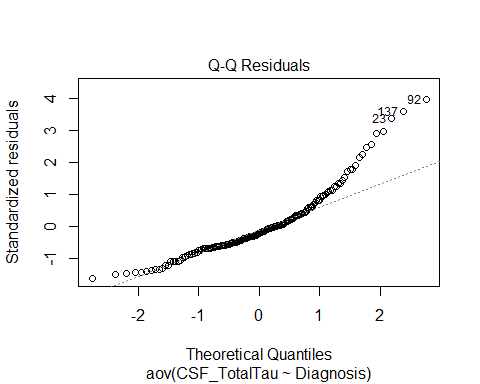

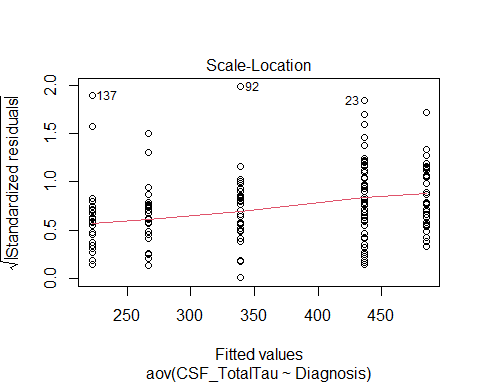

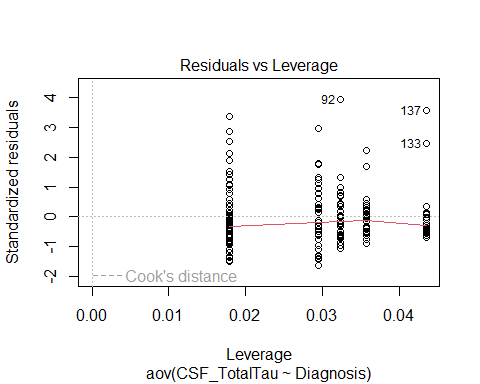


summary(model_ttau)

## Df Sum Sq Mean Sq F value Pr(>F)
## Diagnosis 4 1523484 380871 7.898 7.44e-06 ***
## Residuals 167 8053280 48223
## ---
## Signif. codes: 0 '***' 0.001 '**' 0.01 '*' 0.05 '.' 0.1 ' ' 1
## 15 observations deleted due to missingness

Anova(model_ttau, type = "II")

## Anova Table (Type II tests)
##
## Response: CSF_TotalTau
## Sum Sq Df F value Pr(>F)
## Diagnosis 1523484 4 7.8981 7.444e-06 ***
## Residuals 8053280 167
## ---
## Signif. codes: 0 '***' 0.001 '**' 0.01 '*' 0.05 '.' 0.1 ' ' 1

attach(SNAP25)

## De følgende objekter er maskerede fra SNAP25 (pos = 3):
##
## AB42_Status, AgeAtVisit, CognitiveSyndrome, CSF_ABeta42,
## CSF_PhosphoTau, CSF_SNAP25, CSF_TotalTau, DDBBno, Diagnosis, Group,
## MMSE, Sex, VisitDate
##
## De følgende objekter er maskerede fra SNAP25 (pos = 4):
##
## AB42_Status, AgeAtVisit, CognitiveSyndrome, CSF_ABeta42,
## CSF_PhosphoTau, CSF_SNAP25, CSF_TotalTau, DDBBno, Diagnosis, Group,
## MMSE, Sex, VisitDate

pairwise.t.test(CSF_TotalTau, Diagnosis, p.adj = "none")

##
## Pairwise comparisons using t tests with pooled SD
##
## data: CSF_TotalTau and Diagnosis
##
## AD HC MCI_AD NPH
## HC 0.04914 - - -
## MCI_AD 0.30953 0.00812 - -
## NPH 0.00012 0.05543 1.7e-05 -
## VaD 0.00107 0.21118 0.00014 0.47033
##
## P value adjustment method: none

tapply(CSF_TotalTau, Diagnosis, mean, na.rm=TRUE)

## AD HC MCI_AD NPH VaD
## 436.3929 338.9677 485.0588 222.3913 267.1071

tapply(CSF_TotalTau, Diagnosis, sd, na.rm=TRUE)

## AD HC MCI_AD NPH VaD
## 244.6468 205.4420 236.0683 216.6843 151.9316

# one way ANOVA for p-tau between groups - with post-hoc pairwise t-test:

SNAP25$Diagnosis <- as.factor(SNAP25$Diagnosis)

model_ptau <- aov(CSF_PhosphoTau~Diagnosis,data = SNAP25)

plot(model_ptau)


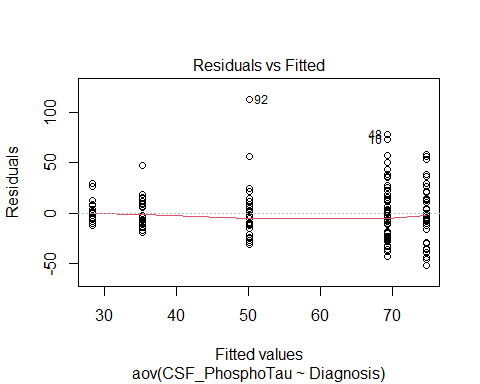

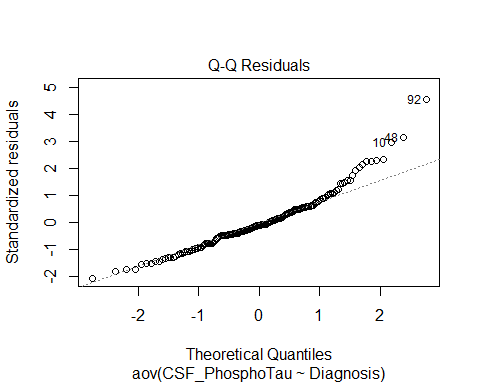

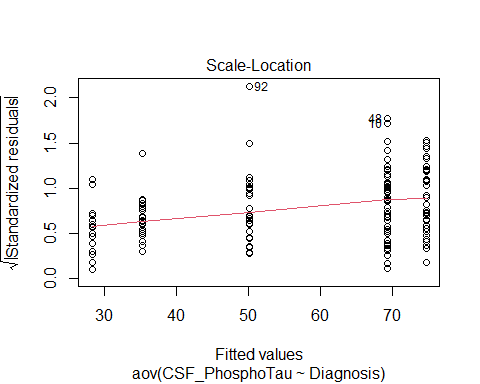

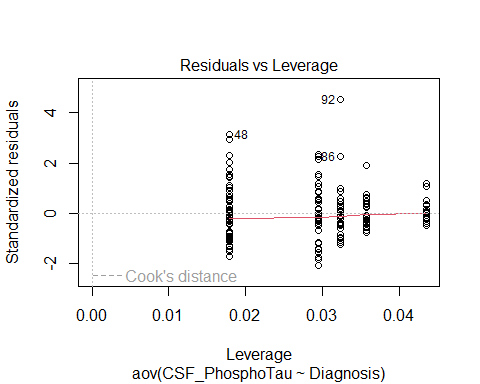


summary(model_ptau)

## Df Sum Sq Mean Sq F value Pr(>F)
## Diagnosis 4 52747 13187 20.56 8.65e-14 ***
## Residuals 167 107129 641
## ---
## Signif. codes: 0 '***' 0.001 '**' 0.01 '*' 0.05 '.' 0.1 ' ' 1
## 15 observations deleted due to missingness

Anova(model_ptau, type = "II")

## Anova Table (Type II tests)
##
## Response: CSF_PhosphoTau
## Sum Sq Df F value Pr(>F)
## Diagnosis 52747 4 20.556 8.647e-14 ***
## Residuals 107129 167
## ---
## Signif. codes: 0 '***' 0.001 '**' 0.01 '*' 0.05 '.' 0.1 ' ' 1

attach(SNAP25)

## De følgende objekter er maskerede fra SNAP25 (pos = 3):
##
## AB42_Status, AgeAtVisit, CognitiveSyndrome, CSF_ABeta42,
## CSF_PhosphoTau, CSF_SNAP25, CSF_TotalTau, DDBBno, Diagnosis, Group,
## MMSE, Sex, VisitDate
##
## De følgende objekter er maskerede fra SNAP25 (pos = 4):
##
## AB42_Status, AgeAtVisit, CognitiveSyndrome, CSF_ABeta42,
## CSF_PhosphoTau, CSF_SNAP25, CSF_TotalTau, DDBBno, Diagnosis, Group,
## MMSE, Sex, VisitDate
##
## De følgende objekter er maskerede fra SNAP25 (pos = 5):
##
## AB42_Status, AgeAtVisit, CognitiveSyndrome, CSF_ABeta42,
## CSF_PhosphoTau, CSF_SNAP25, CSF_TotalTau, DDBBno, Diagnosis, Group,
## MMSE, Sex, VisitDate

pairwise.t.test(CSF_PhosphoTau, Diagnosis, p.adj = "none")

##
## Pairwise comparisons using t tests with pooled SD
##
## data: CSF_PhosphoTau and Diagnosis
##
## AD HC MCI_AD NPH
## HC 0.00089 - - -
## MCI_AD 0.32695 0.00013 - -
## NPH 6.7e-10 0.00198 1.7e-10 -
## VaD 3.1e-08 0.02554 6.8e-09 0.32523
##
## P value adjustment method: none

tapply(CSF_PhosphoTau, Diagnosis, mean, na.rm=TRUE)

## AD HC MCI_AD NPH VaD
## 69.32143 50.12903 74.73529 28.21739 35.25000

tapply(CSF_PhosphoTau, Diagnosis, sd, na.rm=TRUE)

## AD HC MCI_AD NPH VaD
## 28.57533 28.36634 29.40966 11.65394 15.57449

# one way ANOVA for SNAP-25 between groups - with post-hoc pairwise t-test:

SNAP25$Diagnosis <- as.factor(SNAP25$Diagnosis)

model_snap25 <- aov(CSF_SNAP25~Diagnosis+AgeAtVisit,data = SNAP25)

plot(model_snap25)


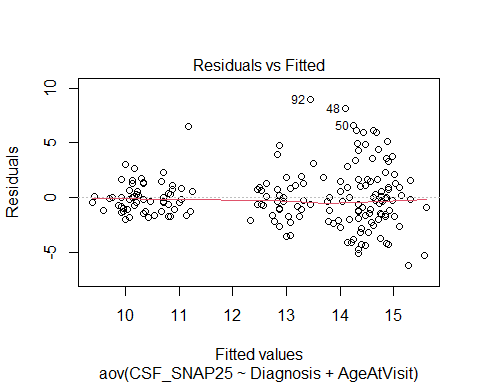

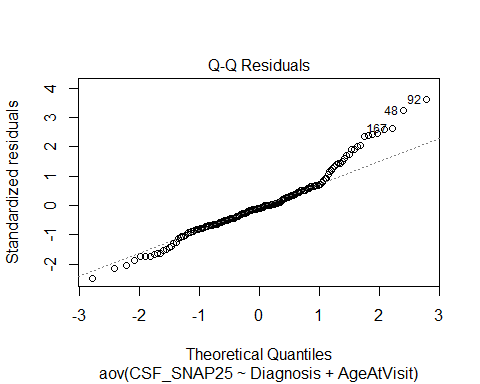

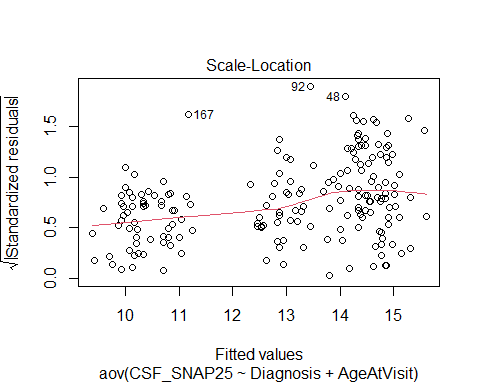

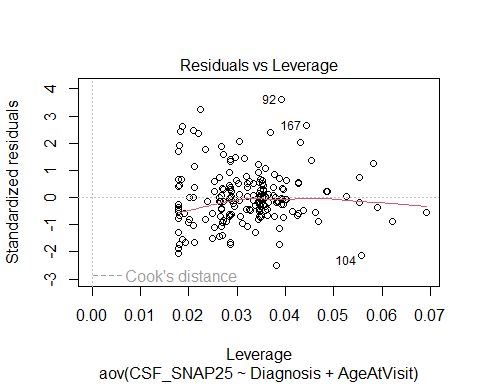


summary(model_snap25)

## Df Sum Sq Mean Sq F value Pr(>F)
## Diagnosis 4 633.8 158.46 24.688 2.54e-16 ***
## AgeAtVisit 1 19.1 19.06 2.969 0.0866 .
## Residuals 181 1161.7 6.42
## ---
## Signif. codes: 0 '***' 0.001 '**' 0.01 '*' 0.05 '.' 0.1 ' ' 1

Anova(model_snap25, type = "II")

## Anova Table (Type II tests)
##
## Response: CSF_SNAP25
## Sum Sq Df F value Pr(>F)
## Diagnosis 650.82 4 25.3495 < 2e-16 ***
## AgeAtVisit 19.06 1 2.9688 0.08659 .
## Residuals 1161.74 181
## ---
## Signif. codes: 0 '***' 0.001 '**' 0.01 '*' 0.05 '.' 0.1 ' ' 1

tapply(CSF_SNAP25, Diagnosis, mean, na.rm=TRUE)

## AD HC MCI_AD NPH VaD
## 14.38929 12.95789 14.84571 10.01034 10.74138

tapply(CSF_SNAP25, Diagnosis, sd, na.rm=TRUE)

## AD HC MCI_AD NPH VaD
## 3.199527 2.406481 2.803500 1.292380 1.787759

#FURTHER ANALYSIS OF SNAP-25 IN THE RETROSPECTIVE CROSS-SECTIONAL STUDY
#Packages:
library(readxl)
library(dplyr)
library(tidyverse)

#Loading of excel file
setwd("P:/RH/NEU/Lukkede Mapper/BiobankCSFProjekt2017/SNAP-25/Statistik_mappe/R/Tværsnit")
SNAP25 <- read_excel('old cohort_with values.xlsx')

#FIRST RESEARCH QUESTION - SNAP-25 ABILITY TO DIFFERENTIATE BETWEEN AD DISEASE STAGES
# Division of the cohort in the desired groups - HC, MCI_AD, AD:

SNAP25$Diagnosis <- as.factor(SNAP25$Diagnosis)
SNAP25_ny <- SNAP25[SNAP25$Diagnosis %in% c('AD', 'MCI_AD', 'HC'),]
SNAP25_ny$Diagnosis <- droplevels(SNAP25_ny$Diagnosis)

# chi squared test for sex distribution between the predefined groups:
tabel <- table(SNAP25_ny$Sex, SNAP25_ny$Diagnosis)
tabel

##
## AD HC MCI_AD
## F 26 16 15
## M 30 22 20

chisq.test(tabel)

##
## Pearson's Chi-squared test
##
## data: tabel
## X-squared = 0.20597, df = 2, p-value = 0.9021

# one way ANOVA for age between the predefined groups:
model_age <- aov(AgeAtVisit~Diagnosis, data = SNAP25_ny)
plot(model_age)


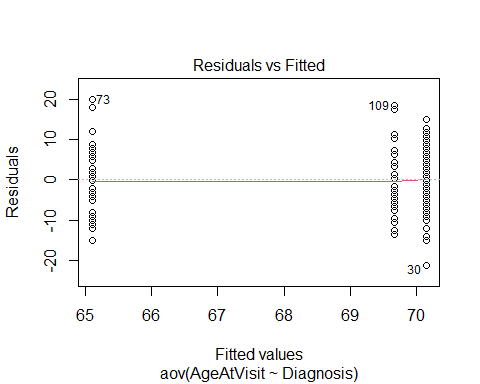

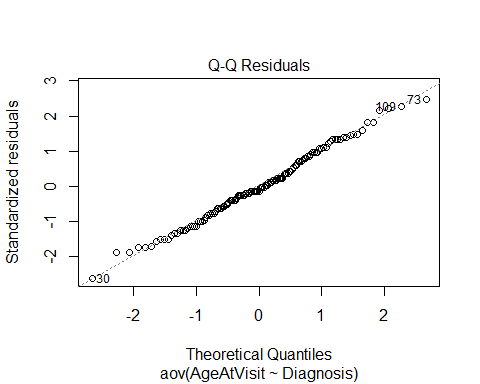

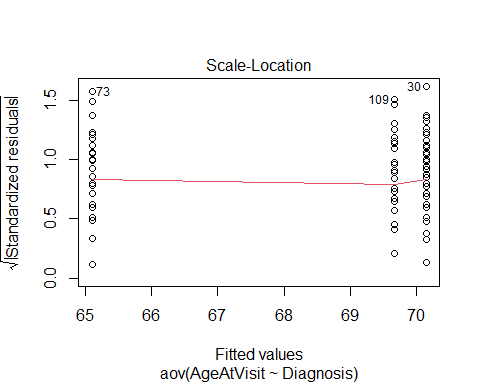

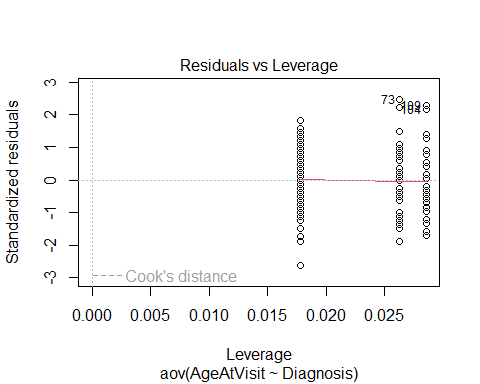


library(car)
Anova(model_age, type = "II") # p-value for the model with a type 2 sum and squares

## Anova Table (Type II tests)
##
## Response: AgeAtVisit
## Sum Sq Df F value Pr(>F)
## Diagnosis 635.8 2 4.7527 0.01023 *
## Residuals 8428.3 126
## ---
## Signif. codes: 0 '***' 0.001 '**' 0.01 '*' 0.05 '.' 0.1 ' ' 1

# an ANCOVA for comparing SNAP-25 between the predefined groups with age as a covariate:

model_SNAP_1 <- aov(log(CSF_SNAP25)~Diagnosis + AgeAtVisit, data = SNAP25_ny)
plot(model_SNAP_1) # control of the model


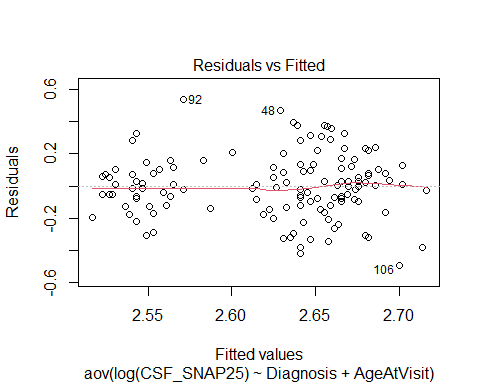

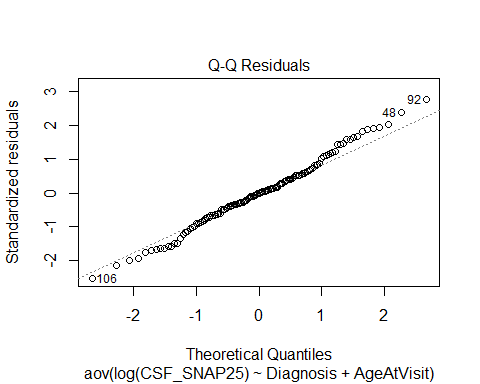

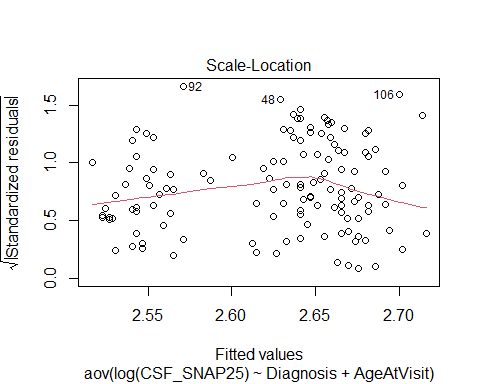

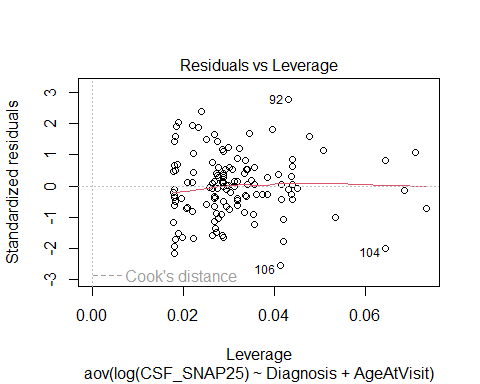


Anova(model_SNAP_1, type = "II")

## Anova Table (Type II tests)
##
## Response: log(CSF_SNAP25)
## Sum Sq Df F value Pr(>F)
## Diagnosis 0.2810 2 3.5532 0.03156 *
## AgeAtVisit 0.0347 1 0.8770 0.35082
## Residuals 4.9425 125
## ---
## Signif. codes: 0 '***' 0.001 '**' 0.01 '*' 0.05 '.' 0.1 ' ' 1

# post-hoc Tukey's test when comparing SNAP-25 between the predefined diagnostic groups:
library(multcomp)

## Warning: pakke 'multcomp' blev bygget under R version 4.3.2

## Indlæser krævet pakke: mvtnorm

## Warning: pakke 'mvtnorm' blev bygget under R version 4.3.2

## Indlæser krævet pakke: survival
## Indlæser krævet pakke: TH.data

## Warning: pakke 'TH.data' blev bygget under R version 4.3.2

## Indlæser krævet pakke: MASS
##
## Vedhæfter pakke: 'MASS'
##
## Det følgende objekt er maskeret fra 'package:dplyr':
##
## select
##
##
## Vedhæfter pakke: 'TH.data'
##
## Det følgende objekt er maskeret fra 'package:MASS':
##
## geyser

attach(model_SNAP_1)
SNAP_mult <- glht(model_SNAP_1, linfct = mcp(Diagnosis = 'Tukey'))
summary(SNAP_mult)

##
## Simultaneous Tests for General Linear Hypotheses
##
## Multiple Comparisons of Means: Tukey Contrasts
##
##
## Fit: aov(formula = log(CSF_SNAP25) ~ Diagnosis + AgeAtVisit, data = SNAP25_ny)
##
## Linear Hypotheses:
## Estimate Std. Error t value Pr(>|t|)
## HC - AD == 0 -0.08612 0.04319 -1.994 0.1175
## MCI_AD - AD == 0 0.03679 0.04286 0.858 0.6668
## MCI_AD - HC == 0 0.12291 0.04762 2.581 0.0293 *
## ---
## Signif. codes: 0 '***' 0.001 '**' 0.01 '*' 0.05 '.' 0.1 ' ' 1
## (Adjusted p values reported -- single-step method)

# CI
confint(SNAP_mult)

##
## Simultaneous Confidence Intervals
##
## Multiple Comparisons of Means: Tukey Contrasts
##
##
## Fit: aov(formula = log(CSF_SNAP25) ~ Diagnosis + AgeAtVisit, data = SNAP25_ny)
##
## Quantile = 2.37
## 95% family-wise confidence level
##
##
## Linear Hypotheses:
## Estimate lwr upr
## HC - AD == 0 -0.08612 -0.18849 0.01625
## MCI_AD - AD == 0 0.03679 -0.06479 0.13836
## MCI_AD - HC == 0 0.12291 0.01005 0.23576

#Bar plot:

order_SNAP <- c("HC", "MCI_AD", "AD")
SNAP25_ny$Diagnosis <- factor(SNAP25_ny$Diagnosis, levels = order_SNAP)
plot(SNAP25_ny$CSF_SNAP25~SNAP25_ny$Diagnosis)


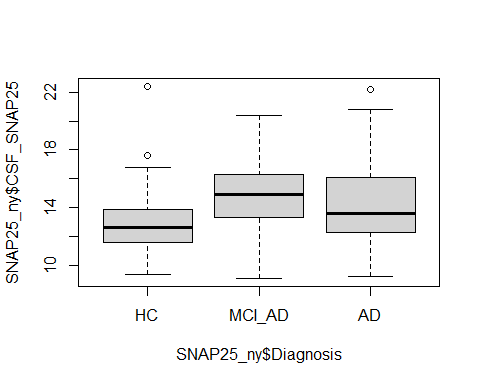


#SECOND RESEARCH QUESTION - SNAP-25 ABILITY TO DIFFERENTIATE BETWEEN PATIENTS WITH OR WITHOUT AD PATHOLOGY
# Division of the cohort in the desired groups - AD Pathology, VaD, NPH:
#1 = AD pathology
#2 = VaD
#3 = NPH

SNAP25$Group <- as.factor(SNAP25$Group)

desired_categories <- c("1", "2", "3")
new_dataset_2 <- subset(SNAP25, SNAP25$Group %in% desired_categories)
new_dataset_2$Group <- droplevels(new_dataset_2$Group)

# chi squared test for sex distribution between the predefined groups:
tabel <- table(new_dataset_2$Sex, new_dataset_2$Group)
tabel

##
## 1 2 3
## F 41 6 10
## M 50 23 19

chisq.test(tabel)

##
## Pearson's Chi-squared test
##
## data: tabel
## X-squared = 5.7442, df = 2, p-value = 0.05658

# one way ANOVA for age between the predefined groups:
model_age_a2 <- aov(AgeAtVisit~Group, data = new_dataset_2)
plot(model_age_a2)


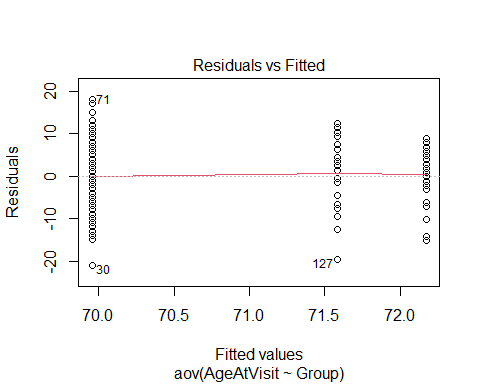

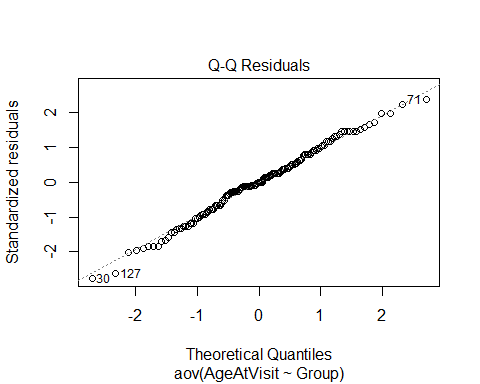

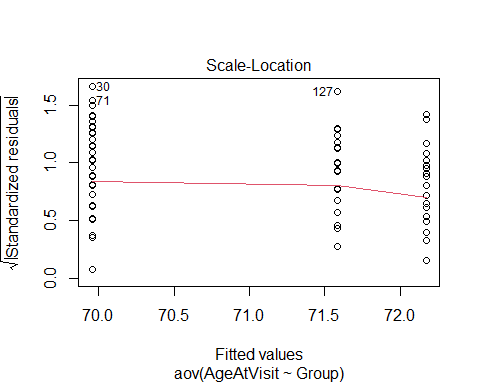

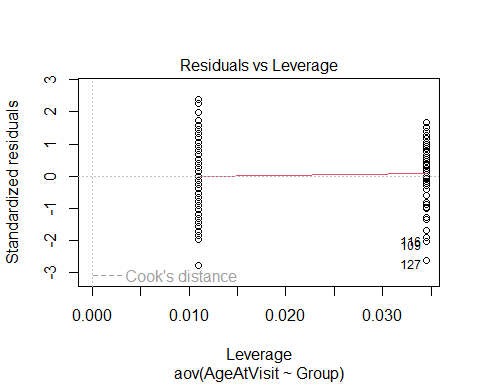


Anova(model_age_a2, type = "II") # p-value for the model with a type 2 sum and squares

## Anova Table (Type II tests)
##
## Response: AgeAtVisit
## Sum Sq Df F value Pr(>F)
## Group 136 2 1.1691 0.3136
## Residuals 8493 146

# an ANCOVA for comparing SNAP-25 between the predefined groups:
model_SNAP_2 <- aov(log(CSF_SNAP25)~Group, data = new_dataset_2)
plot(model_SNAP_2) # control of model


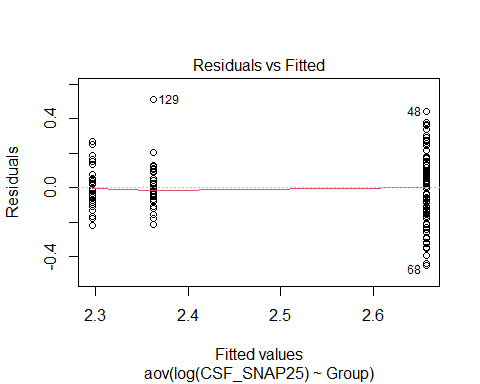

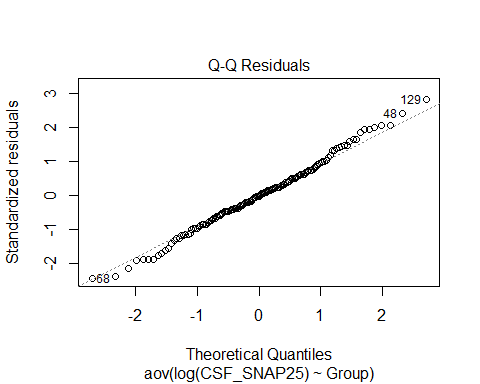

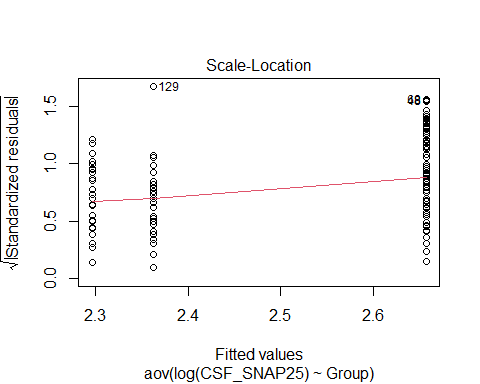

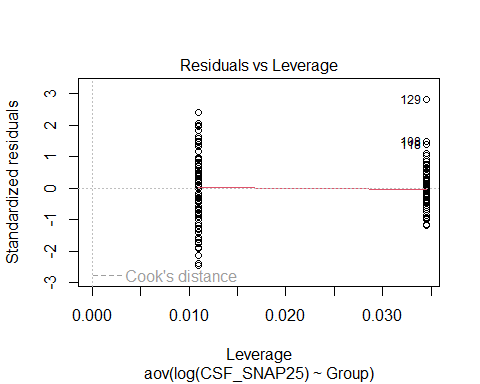


Anova(model_SNAP_2, type = "II")

## Anova Table (Type II tests)
##
## Response: log(CSF_SNAP25)
## Sum Sq Df F value Pr(>F)
## Group 3.8719 2 56.388 < 2.2e-16 ***
## Residuals 5.0125 146
## ---
## Signif. codes: 0 '***' 0.001 '**' 0.01 '*' 0.05 '.' 0.1 ' ' 1

# Post-hoc Tukey's test comparing SNAP-25 between the predefined groups:
SNAP_mult_2 <- glht(model_SNAP_2, linfct = mcp(Group = 'Tukey'))
summary(SNAP_mult_2)

##
## Simultaneous Tests for General Linear Hypotheses
##
## Multiple Comparisons of Means: Tukey Contrasts
##
##
## Fit: aov(formula = log(CSF_SNAP25) ~ Group, data = new_dataset_2)
##
## Linear Hypotheses:
## Estimate Std. Error t value Pr(>|t|)
## 2 - 1 == 0 -0.29454 0.03951 -7.455 <1e-04 ***
## 3 - 1 == 0 -0.36116 0.03951 -9.141 <1e-04 ***
## 3 - 2 == 0 -0.06662 0.04866 -1.369 0.356
## ---
## Signif. codes: 0 '***' 0.001 '**' 0.01 '*' 0.05 '.' 0.1 ' ' 1
## (Adjusted p values reported -- single-step method)

plot(log(new_dataset_2$CSF_SNAP25)~new_dataset_2$Group)


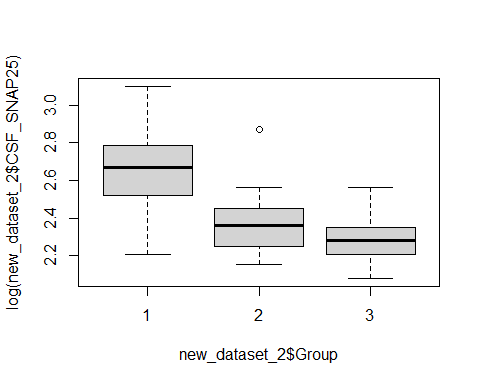


#confidensinterval:
confint(SNAP_mult_2)

##
## Simultaneous Confidence Intervals
##
## Multiple Comparisons of Means: Tukey Contrasts
##
##
## Fit: aov(formula = log(CSF_SNAP25) ~ Group, data = new_dataset_2)
##
## Quantile = 2.3606
## 95% family-wise confidence level
##
##
## Linear Hypotheses:
## Estimate lwr upr
## 2 - 1 == 0 -0.29454 -0.38781 -0.20127
## 3 - 1 == 0 -0.36116 -0.45443 -0.26789
## 3 - 2 == 0 -0.06662 -0.18149 0.04825

#ASSOCIATION OF SNAP-25 WITH TAU PROTEINS:
# Linear regression between SNAP-25 and p-tau (WITHOUR LOG):

linear_model <- lm(CSF_SNAP25~CSF_PhosphoTau, data = SNAP25)
summary(linear_model)

##
## Call:
## lm(formula = CSF_SNAP25 ~ CSF_PhosphoTau, data = SNAP25)
##
## Residuals:
## Min 1Q Median 3Q Max
## -3.5966 -0.7889 -0.0792 0.7953 4.4114
##
## Coefficients:
## Estimate Std. Error t value Pr(>|t|)
## (Intercept) 7.696904 0.193178 39.84 <2e-16 ***
## CSF_PhosphoTau 0.095291 0.003034 31.40 <2e-16 ***
## ---
## Signif. codes: 0 '***' 0.001 '**' 0.01 '*' 0.05 '.' 0.1 ' ' 1
##
## Residual standard error: 1.213 on 170 degrees of freedom
## (15 observations deleted due to missingness)
## Multiple R-squared: 0.853, Adjusted R-squared: 0.8521
## F-statistic: 986.2 on 1 and 170 DF, p-value: < 2.2e-16

plot(linear_model)


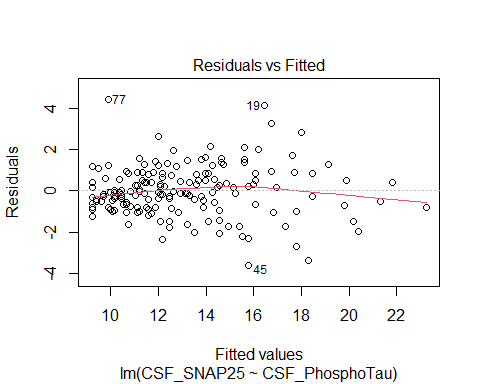

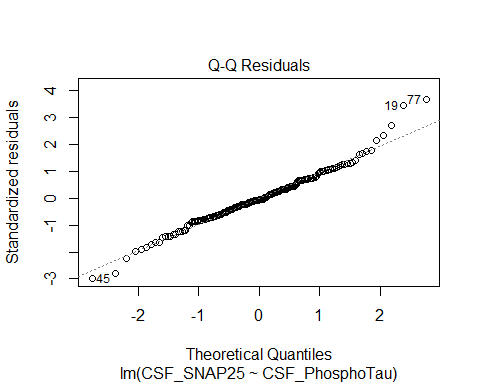

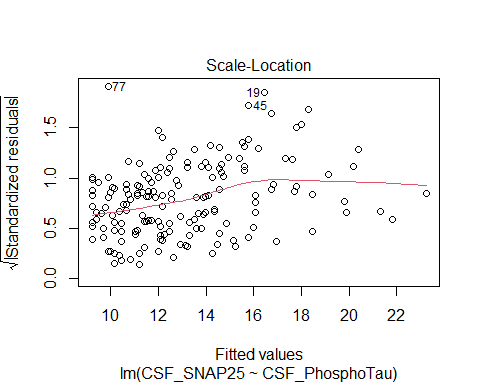

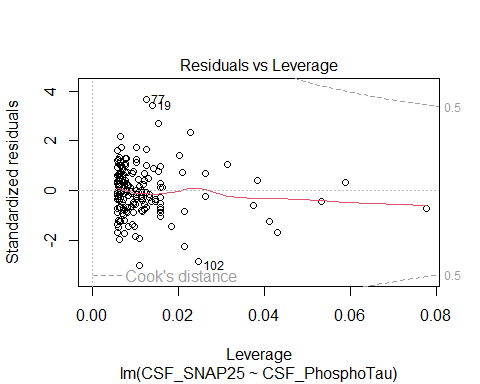


# Linear regression between SNAP-25 and p-tau (WITH LOG):

linear_model_ptau <- lm(log(CSF_SNAP25)~CSF_PhosphoTau, data = SNAP25)
summary(linear_model_ptau)

##
## Call:
## lm(formula = log(CSF_SNAP25) ~ CSF_PhosphoTau, data = SNAP25)
##
## Residuals:
## Min 1Q Median 3Q Max
## -0.23932 -0.07128 0.00267 0.06512 0.34862
##
## Coefficients:
## Estimate Std. Error t value Pr(>|t|)
## (Intercept) 2.152448 0.015215 141.47 <2e-16 ***
## CSF_PhosphoTau 0.006921 0.000239 28.96 <2e-16 ***
## ---
## Signif. codes: 0 '***' 0.001 '**' 0.01 '*' 0.05 '.' 0.1 ' ' 1
##
## Residual standard error: 0.09556 on 170 degrees of freedom
## (15 observations deleted due to missingness)
## Multiple R-squared: 0.8315, Adjusted R-squared: 0.8305
## F-statistic: 838.7 on 1 and 170 DF, p-value: < 2.2e-16

plot(linear_model_ptau)


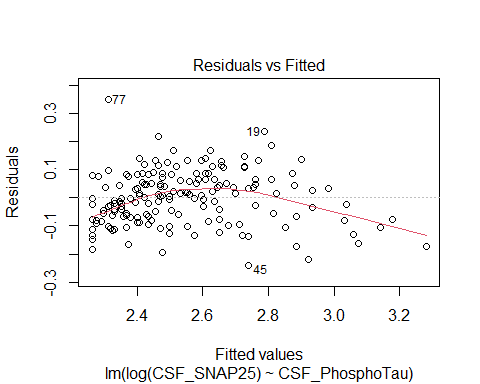

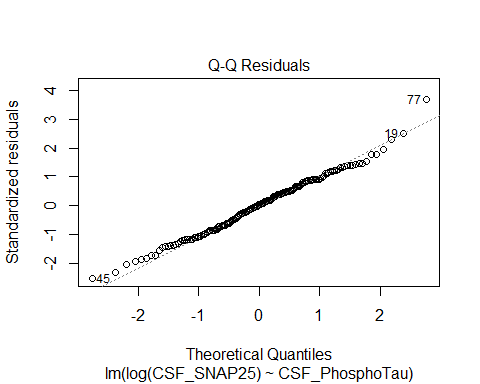

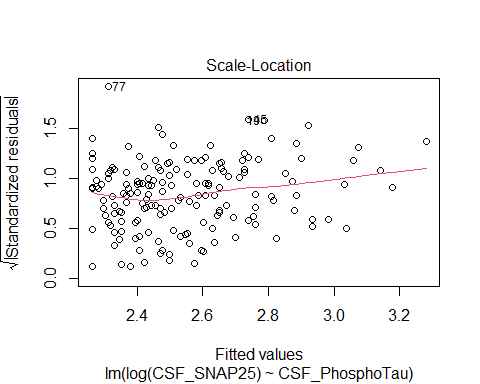

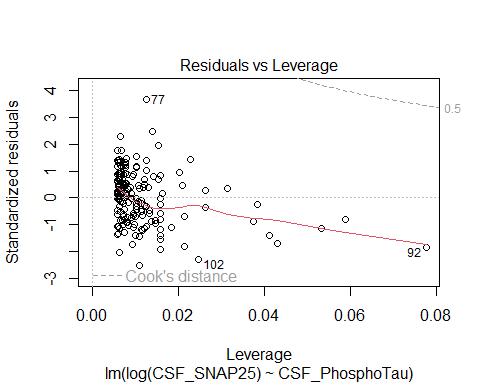


# Linear regression between SNAP-25 and t-tau (WITHOUT LOG):

linear_model <- lm(log(CSF_SNAP25)~CSF_TotalTau, data = SNAP25)
summary(linear_model)

##
## Call:
## lm(formula = log(CSF_SNAP25) ~ CSF_TotalTau, data = SNAP25)
##
## Residuals:
## Min 1Q Median 3Q Max
## -0.68547 -0.08214 0.00126 0.09717 0.59008
##
## Coefficients:
## Estimate Std. Error t value Pr(>|t|)
## (Intercept) 2.274e+00 2.279e-02 99.77 <2e-16 ***
## CSF_TotalTau 7.122e-04 5.171e-05 13.77 <2e-16 ***
## ---
## Signif. codes: 0 '***' 0.001 '**' 0.01 '*' 0.05 '.' 0.1 ' ' 1
##
## Residual standard error: 0.16 on 170 degrees of freedom
## (15 observations deleted due to missingness)
## Multiple R-squared: 0.5273, Adjusted R-squared: 0.5245
## F-statistic: 189.7 on 1 and 170 DF, p-value: < 2.2e-16

plot(linear_model)


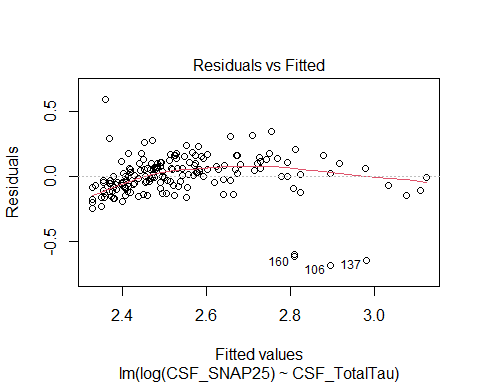

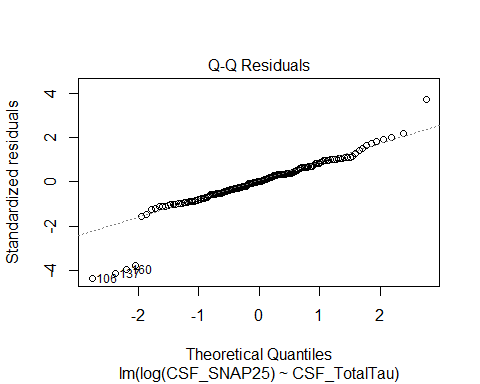

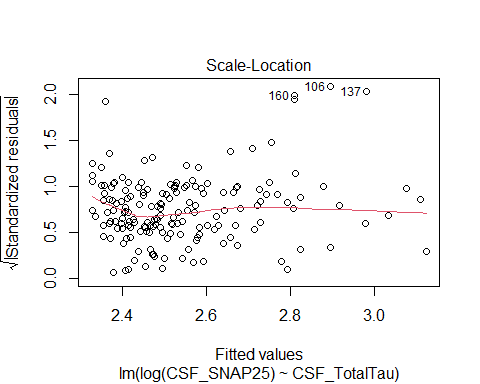

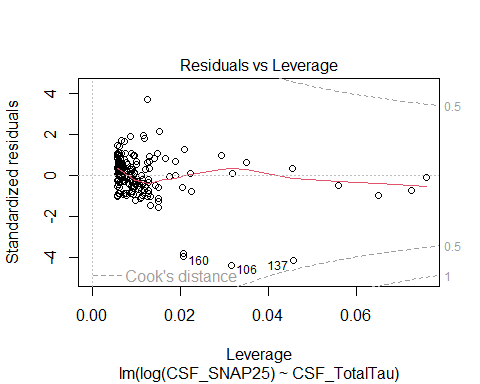


# Linear regression between SNAP-25 and t-tau (WITH LOG):

linear_model_ttau <- lm(log(CSF_SNAP25)~CSF_TotalTau, data = SNAP25)
summary(linear_model_ttau)

##
## Call:
## lm(formula = log(CSF_SNAP25) ~ CSF_TotalTau, data = SNAP25)
##
## Residuals:
## Min 1Q Median 3Q Max
## -0.68547 -0.08214 0.00126 0.09717 0.59008
##
## Coefficients:
## Estimate Std. Error t value Pr(>|t|)
## (Intercept) 2.274e+00 2.279e-02 99.77 <2e-16 ***
## CSF_TotalTau 7.122e-04 5.171e-05 13.77 <2e-16 ***
## ---
## Signif. codes: 0 '***' 0.001 '**' 0.01 '*' 0.05 '.' 0.1 ' ' 1
##
## Residual standard error: 0.16 on 170 degrees of freedom
## (15 observations deleted due to missingness)
## Multiple R-squared: 0.5273, Adjusted R-squared: 0.5245
## F-statistic: 189.7 on 1 and 170 DF, p-value: < 2.2e-16

plot(linear_model_ttau)


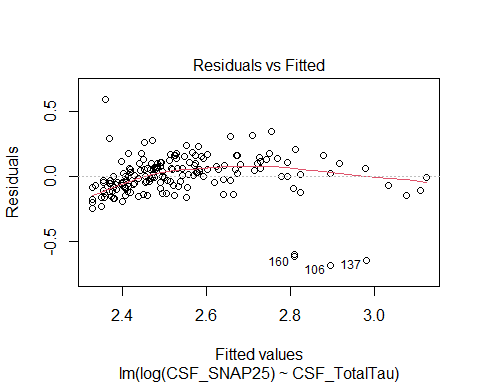

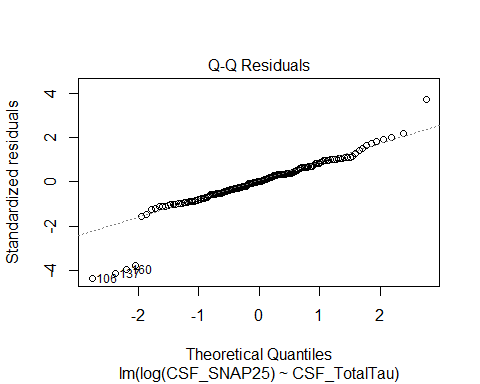

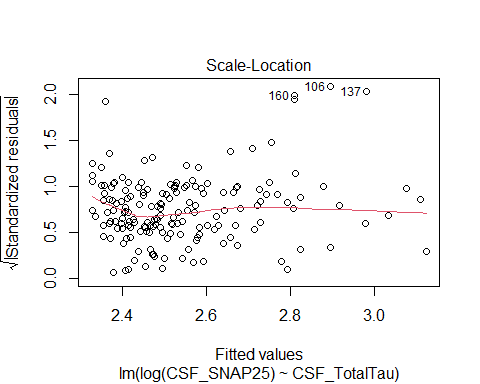

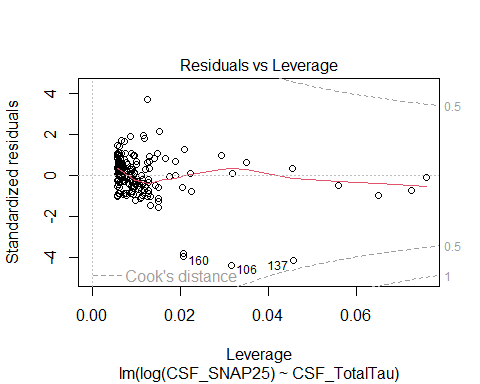


# Association between SNAP-25 and p-tau
library(ggplot2)
ggplot(SNAP25, aes(x=CSF_SNAP25, y=CSF_PhosphoTau, color = Diagnosis)) +
 geom_point(size = 3) +
 geom_smooth(method=lm , color="red", fill="#69b3a2", se=TRUE) +
 xlab("CSF SNAP25 [pM]") + ylab("CSF ptau [pg/ml]")

## `geom_smooth()` using formula = 'y ~ x'

## Warning: Removed 15 rows containing non-finite values (`stat_smooth()`).

## Warning: Removed 15 rows containing missing values (`geom_point()`).


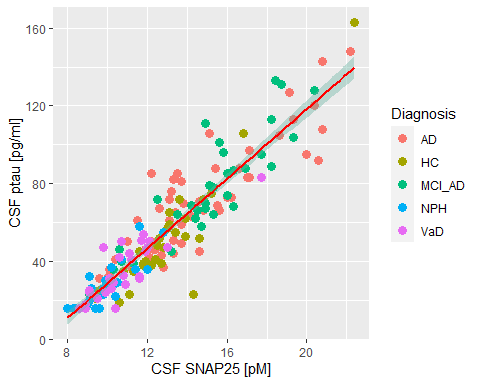


# Association between SNAP-25 and t-tau
library(ggplot2)
ggplot(SNAP25, aes(x=CSF_SNAP25, y=CSF_TotalTau, color = Diagnosis)) +
 geom_point(size = 3) +
 geom_smooth(method=lm , color="red", fill="#69b3a2", se=TRUE) +
 xlab("CSF SNAP25 [pM]") + ylab("CSF total tau [pg/ml]")

## `geom_smooth()` using formula = 'y ~ x'

## Warning: Removed 15 rows containing non-finite values (`stat_smooth()`).
## Removed 15 rows containing missing values (`geom_point()`).


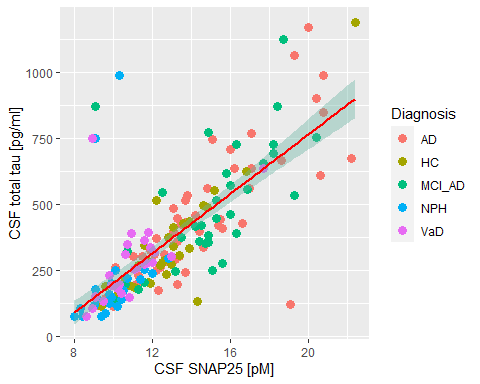


##
##
##
## Prospective Cohort
##
##
##

# PATIENT CHARACTERISTICS FOR THE PROSPECTIVE STUDY
# Packages:
library(car)
library(readxl)
library(dplyr)
library(tidyverse)

#Loading of Excel file
setwd("P:/RH/NEU/Lukkede Mapper/BiobankCSFProjekt2017/SNAP-25/Statistik_mappe/R/Prospektivt")
cohort <- read_excel('Prospective study_demografitabel.xlsx')

## New names:
## • `` -> `...12`

# chi squared test for sex distribution between the groups

table1 <- table(cohort$Sex, cohort$Diagnosis)
table1

##
## AD HC MCI_AD Non-AD-ND Non_ND_D
## F 31 5 8 7 10
## M 19 9 2 11 32

chisq.test(table1)

## Warning in chisq.test(table1): Chi-squared approximation may be incorrect

##
## Pearson's Chi-squared test
##
## data: table1
## X-squared = 19.114, df = 4, p-value = 0.0007464

# chi squared test for amyloid status between groups

table2 <- table(cohort$AB42_status, cohort$Diagnosis)
table2

##
## AD HC MCI_AD Non-AD-ND Non_ND_D
## AB_negative 3 4 1 11 20
## AB_positive 47 2 9 7 22

chisq.test(table2)

## Warning in chisq.test(table2): Chi-squared approximation may be incorrect

##
## Pearson's Chi-squared test
##
## data: table2
## X-squared = 33.321, df = 4, p-value = 1.027e-06

# one way ANOVA for age between groups - with post-hoc pairwise t-test:

cohort$Diagnosis <- as.factor(cohort$Diagnosis)

model_age <- aov(Age~Diagnosis,data = cohort)

plot(model_age)


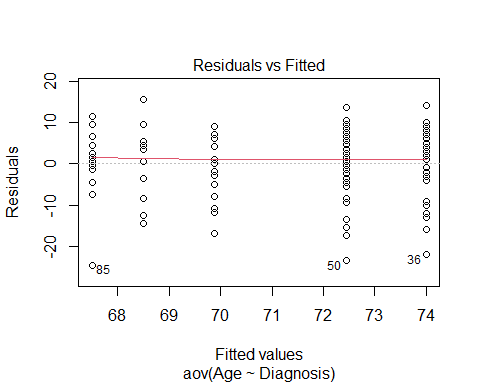

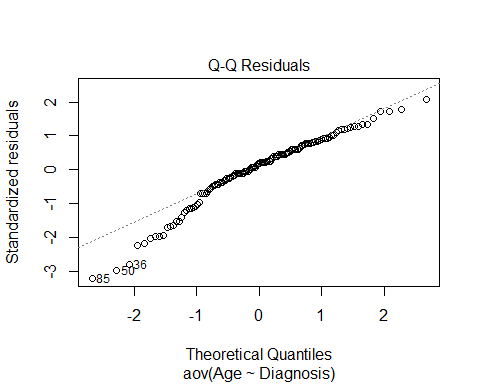

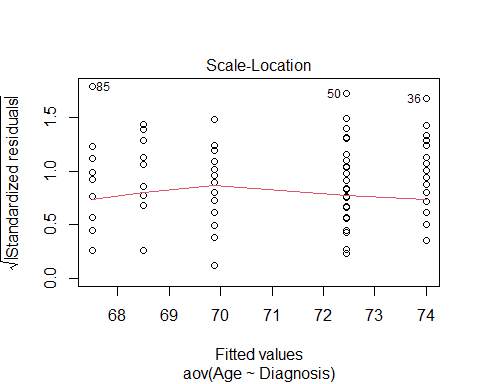

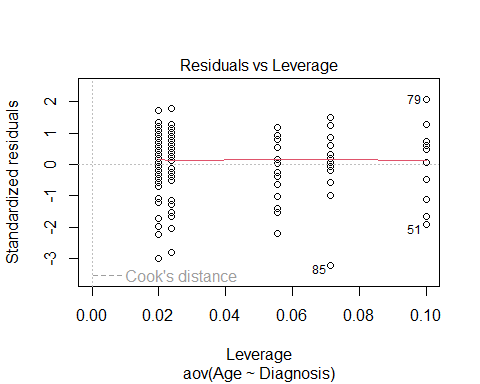


summary(model_age)

## Df Sum Sq Mean Sq F value Pr(>F)
## Diagnosis 4 657 164.3 2.633 0.0372 *
## Residuals 129 8050 62.4
## ---
## Signif. codes: 0 '***' 0.001 '**' 0.01 '*' 0.05 '.' 0.1 ' ' 1

Anova(model_age, type = "II")

## Warning in printHypothesis(L, rhs, names(b)): one or more coefficients in the hypothesis include
## arithmetic operators in their names;
## the printed representation of the hypothesis will be omitted

## Anova Table (Type II tests)
##
## Response: Age
## Sum Sq Df F value Pr(>F)
## Diagnosis 657.2 4 2.6328 0.03717 *
## Residuals 8050.1 129
## ---
## Signif. codes: 0 '***' 0.001 '**' 0.01 '*' 0.05 '.' 0.1 ' ' 1

summary(model_age)

## Df Sum Sq Mean Sq F value Pr(>F)
## Diagnosis 4 657 164.3 2.633 0.0372 *
## Residuals 129 8050 62.4
## ---
## Signif. codes: 0 '***' 0.001 '**' 0.01 '*' 0.05 '.' 0.1 ' ' 1

attach(cohort)

## De følgende objekter er maskerede fra SNAP25 (pos = 9):
##
## CSF_ABeta42, CSF_PhosphoTau, CSF_SNAP25, CSF_TotalTau, Diagnosis,
## MMSE, Sex
##
## De følgende objekter er maskerede fra SNAP25 (pos = 10):
##
## CSF_ABeta42, CSF_PhosphoTau, CSF_SNAP25, CSF_TotalTau, Diagnosis,
## MMSE, Sex
##
## De følgende objekter er maskerede fra SNAP25 (pos = 11):
##
## CSF_ABeta42, CSF_PhosphoTau, CSF_SNAP25, CSF_TotalTau, Diagnosis,
## MMSE, Sex
##
## De følgende objekter er maskerede fra SNAP25 (pos = 12):
##
## CSF_ABeta42, CSF_PhosphoTau, CSF_SNAP25, CSF_TotalTau, Diagnosis,
## MMSE, Sex

pairwise.t.test(Age, Diagnosis, p.adj = "none")

##
## Pairwise comparisons using t tests with pooled SD
##
## data: Age and Diagnosis
##
## AD HC MCI_AD Non-AD-ND
## HC 0.0406 - - -
## MCI_AD 0.1523 0.7603 - -
## Non-AD-ND 0.2422 0.3977 0.6565 -
## Non_ND_D 0.3472 0.0087 0.0500 0.0670
##
## P value adjustment method: none

tapply(Age, Diagnosis, mean, na.rm=TRUE)

## AD HC MCI_AD Non-AD-ND Non_ND_D
## 72.44000 67.50000 68.50000 69.88889 74.00000

tapply(Age, Diagnosis, sd, na.rm=TRUE)

## AD HC MCI_AD Non-AD-ND Non_ND_D
## 7.825025 8.662474 9.686761 7.873178 7.285134

# one way ANOVA for MMSE between groups - with post-hoc pairwise t-test:

cohort$Diagnosis <- as.factor(cohort$Diagnosis)

model_mmse <- aov(MMSE~Diagnosis,data = cohort)

plot(model_mmse)


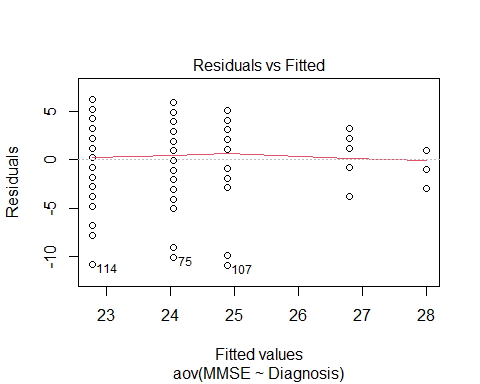

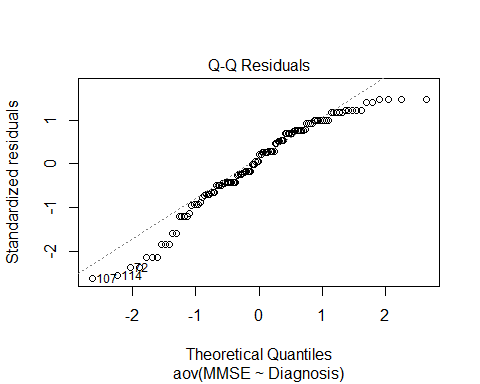

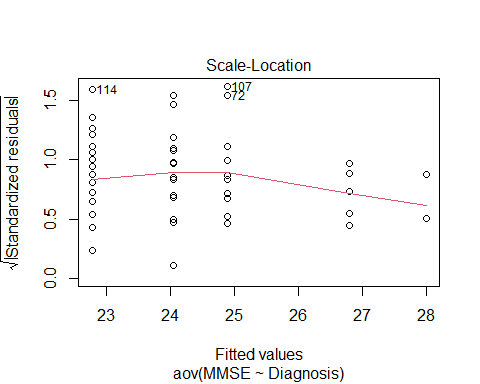

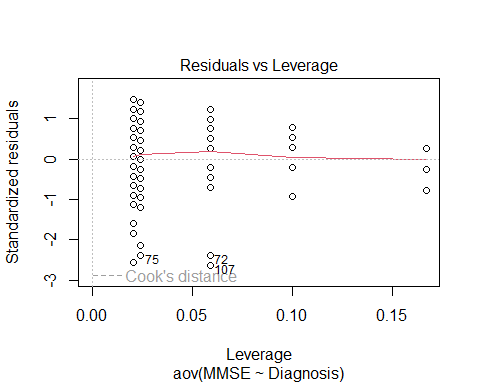


summary(model_mmse)

## Df Sum Sq Mean Sq F value Pr(>F)
## Diagnosis 4 259.4 64.86 3.546 0.00907 **
## Residuals 117 2139.7 18.29
## ---
## Signif. codes: 0 '***' 0.001 '**' 0.01 '*' 0.05 '.' 0.1 ' ' 1
## 12 observations deleted due to missingness

summary(model_mmse)

## Df Sum Sq Mean Sq F value Pr(>F)
## Diagnosis 4 259.4 64.86 3.546 0.00907 **
## Residuals 117 2139.7 18.29
## ---
## Signif. codes: 0 '***' 0.001 '**' 0.01 '*' 0.05 '.' 0.1 ' ' 1
## 12 observations deleted due to missingness

Anova(model_mmse, type = "II")

## Warning in printHypothesis(L, rhs, names(b)): one or more coefficients in the hypothesis include
## arithmetic operators in their names;
## the printed representation of the hypothesis will be omitted

## Anova Table (Type II tests)
##
## Response: MMSE
## Sum Sq Df F value Pr(>F)
## Diagnosis 259.43 4 3.5464 0.009065 **
## Residuals 2139.75 117
## ---
## Signif. codes: 0 '***' 0.001 '**' 0.01 '*' 0.05 '.' 0.1 ' ' 1

attach(cohort)

## De følgende objekter er maskerede fra cohort (pos = 3):
##
## ...12, AB42_status, Age, CSF_ABeta42, CSF_PhosphoTau, CSF_SNAP25,
## CSF_TotalTau, Diagnosis, Groups, MMSE, Sample ID, Sex
##
## De følgende objekter er maskerede fra SNAP25 (pos = 10):
##
## CSF_ABeta42, CSF_PhosphoTau, CSF_SNAP25, CSF_TotalTau, Diagnosis,
## MMSE, Sex
##
## De følgende objekter er maskerede fra SNAP25 (pos = 11):
##
## CSF_ABeta42, CSF_PhosphoTau, CSF_SNAP25, CSF_TotalTau, Diagnosis,
## MMSE, Sex
##
## De følgende objekter er maskerede fra SNAP25 (pos = 12):
##
## CSF_ABeta42, CSF_PhosphoTau, CSF_SNAP25, CSF_TotalTau, Diagnosis,
## MMSE, Sex
##
## De følgende objekter er maskerede fra SNAP25 (pos = 13):
##
## CSF_ABeta42, CSF_PhosphoTau, CSF_SNAP25, CSF_TotalTau, Diagnosis,
## MMSE, Sex

pairwise.t.test(MMSE, Diagnosis, p.adj = "none")

##
## Pairwise comparisons using t tests with pooled SD
##
## data: MMSE and Diagnosis
##
## AD HC MCI_AD Non-AD-ND
## HC 0.0056 - - -
## MCI_AD 0.0077 0.5879 - -
## Non-AD-ND 0.0828 0.1274 0.2628 -
## Non_ND_D 0.1626 0.0367 0.0707 0.5006
##
## P value adjustment method: none

tapply(MMSE, Diagnosis, mean, na.rm=TRUE)

## AD HC MCI_AD Non-AD-ND Non_ND_D
## 22.77083 28.00000 26.80000 24.88235 24.04878

tapply(MMSE, Diagnosis, sd, na.rm=TRUE)

## AD HC MCI_AD Non-AD-ND Non_ND_D
## 4.248853 1.673320 2.820559 4.960876 4.505281

# one way ANOVA for t-tau between groups - with post-hoc pairwise t-test:

cohort$Diagnosis <- as.factor(cohort$Diagnosis)

model_ttau <- aov(CSF_TotalTau~Diagnosis,data = cohort)

plot(model_ttau)


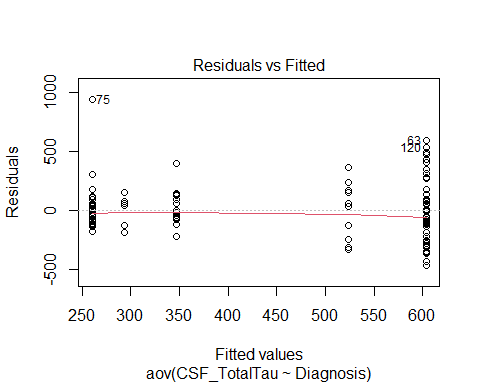

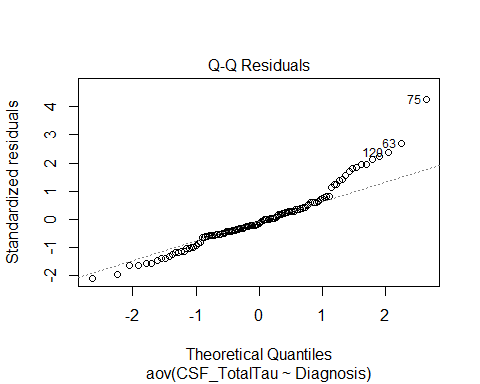

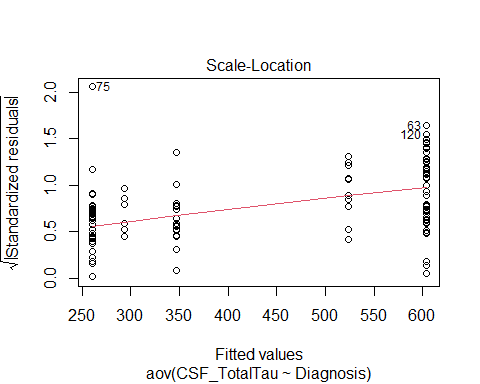

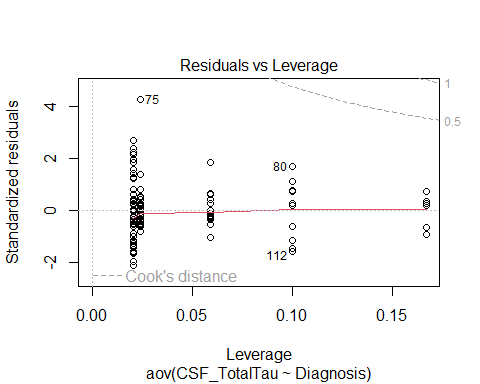


summary(model_ttau)

## Df Sum Sq Mean Sq F value Pr(>F)
## Diagnosis 4 2976100 744025 14.81 7.81e-10 ***
## Residuals 118 5928436 50241
## ---
## Signif. codes: 0 '***' 0.001 '**' 0.01 '*' 0.05 '.' 0.1 ' ' 1
## 11 observations deleted due to missingness

Anova(model_ttau, type = "II")

## Warning in printHypothesis(L, rhs, names(b)): one or more coefficients in the hypothesis include
## arithmetic operators in their names;
## the printed representation of the hypothesis will be omitted

## Anova Table (Type II tests)
##
## Response: CSF_TotalTau
## Sum Sq Df F value Pr(>F)
## Diagnosis 2976100 4 14.809 7.812e-10 ***
## Residuals 5928436 118
## ---
## Signif. codes: 0 '***' 0.001 '**' 0.01 '*' 0.05 '.' 0.1 ' ' 1

attach(cohort)

## De følgende objekter er maskerede fra cohort (pos = 3):
##
## ...12, AB42_status, Age, CSF_ABeta42, CSF_PhosphoTau, CSF_SNAP25,
## CSF_TotalTau, Diagnosis, Groups, MMSE, Sample ID, Sex
##
## De følgende objekter er maskerede fra cohort (pos = 4):
##
## ...12, AB42_status, Age, CSF_ABeta42, CSF_PhosphoTau, CSF_SNAP25,
## CSF_TotalTau, Diagnosis, Groups, MMSE, Sample ID, Sex
##
## De følgende objekter er maskerede fra SNAP25 (pos = 11):
##
## CSF_ABeta42, CSF_PhosphoTau, CSF_SNAP25, CSF_TotalTau, Diagnosis,
## MMSE, Sex
##
## De følgende objekter er maskerede fra SNAP25 (pos = 12):
##
## CSF_ABeta42, CSF_PhosphoTau, CSF_SNAP25, CSF_TotalTau, Diagnosis,
## MMSE, Sex
##
## De følgende objekter er maskerede fra SNAP25 (pos = 13):
##
## CSF_ABeta42, CSF_PhosphoTau, CSF_SNAP25, CSF_TotalTau, Diagnosis,
## MMSE, Sex
##
## De følgende objekter er maskerede fra SNAP25 (pos = 14):
##
## CSF_ABeta42, CSF_PhosphoTau, CSF_SNAP25, CSF_TotalTau, Diagnosis,
## MMSE, Sex

pairwise.t.test(CSF_TotalTau, Diagnosis, p.adj = "none")

##
## Pairwise comparisons using t tests with pooled SD
##
## data: CSF_TotalTau and Diagnosis
##
## AD HC MCI_AD Non-AD-ND
## HC 0.0018 - - -
## MCI_AD 0.3071 0.0494 - -
## Non-AD-ND 8.4e-05 0.6222 0.0495 -
## Non_ND_D 5.0e-11 0.7310 0.0011 0.1843
##
## P value adjustment method: none

tapply(CSF_TotalTau, Diagnosis, mean, na.rm=TRUE)

## AD HC MCI_AD Non-AD-ND Non_ND_D
## 603.4898 293.8333 523.7000 346.4118 260.0732

tapply(CSF_TotalTau, Diagnosis, sd, na.rm=TRUE)

## AD HC MCI_AD Non-AD-ND Non_ND_D
## 277.0854 131.1220 241.8962 149.9067 178.2576

# one way ANOVA for p-tau between groups - with post-hoc pairwise t-test:

cohort$Diagnosis <- as.factor(cohort$Diagnosis)

model_ptau <- aov(CSF_PhosphoTau~Diagnosis,data = cohort)

plot(model_ptau)


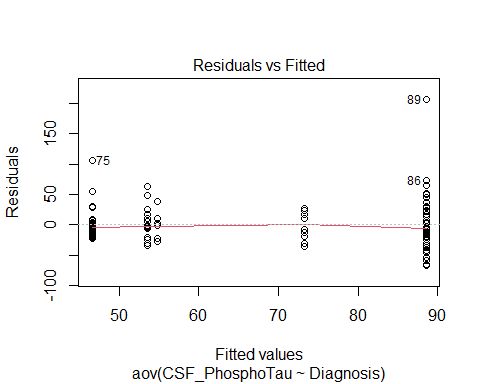

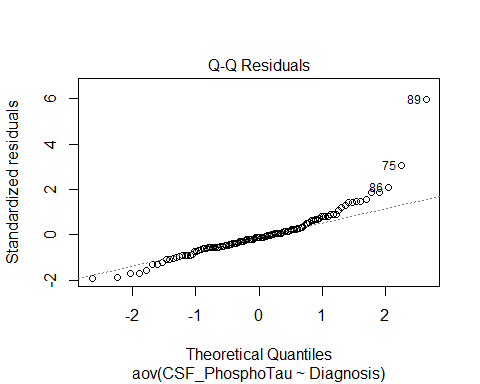

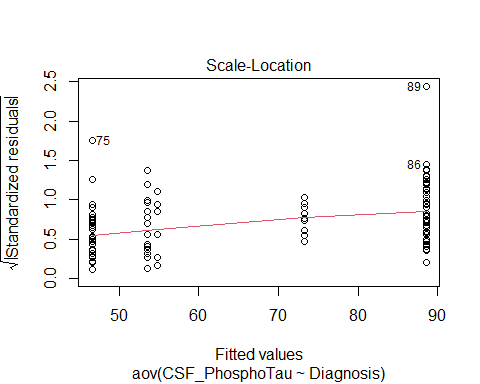

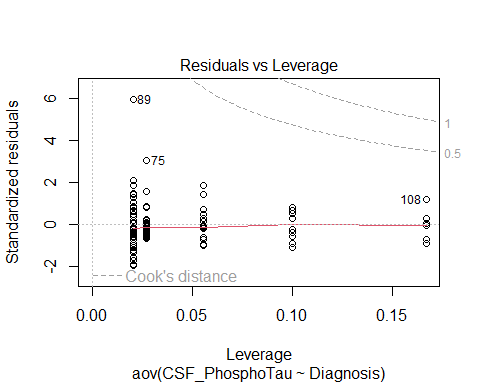


summary(model_ptau)

## Df Sum Sq Mean Sq F value Pr(>F)
## Diagnosis 4 42699 10675 8.7 3.59e-06 ***
## Residuals 115 141096 1227
## ---
## Signif. codes: 0 '***' 0.001 '**' 0.01 '*' 0.05 '.' 0.1 ' ' 1
## 14 observations deleted due to missingness

Anova(model_ptau, type = "II")

## Warning in printHypothesis(L, rhs, names(b)): one or more coefficients in the hypothesis include
## arithmetic operators in their names;
## the printed representation of the hypothesis will be omitted

## Anova Table (Type II tests)
##
## Response: CSF_PhosphoTau
## Sum Sq Df F value Pr(>F)
## Diagnosis 42699 4 8.7004 3.59e-06 ***
## Residuals 141096 115
## ---
## Signif. codes: 0 '***' 0.001 '**' 0.01 '*' 0.05 '.' 0.1 ' ' 1

attach(cohort)

## De følgende objekter er maskerede fra cohort (pos = 3):
##
## ...12, AB42_status, Age, CSF_ABeta42, CSF_PhosphoTau, CSF_SNAP25,
## CSF_TotalTau, Diagnosis, Groups, MMSE, Sample ID, Sex
##
## De følgende objekter er maskerede fra cohort (pos = 4):
##
## ...12, AB42_status, Age, CSF_ABeta42, CSF_PhosphoTau, CSF_SNAP25,
## CSF_TotalTau, Diagnosis, Groups, MMSE, Sample ID, Sex
##
## De følgende objekter er maskerede fra cohort (pos = 5):
##
## ...12, AB42_status, Age, CSF_ABeta42, CSF_PhosphoTau, CSF_SNAP25,
## CSF_TotalTau, Diagnosis, Groups, MMSE, Sample ID, Sex
##
## De følgende objekter er maskerede fra SNAP25 (pos = 12):
##
## CSF_ABeta42, CSF_PhosphoTau, CSF_SNAP25, CSF_TotalTau, Diagnosis,
## MMSE, Sex
##
## De følgende objekter er maskerede fra SNAP25 (pos = 13):
##
## CSF_ABeta42, CSF_PhosphoTau, CSF_SNAP25, CSF_TotalTau, Diagnosis,
## MMSE, Sex
##
## De følgende objekter er maskerede fra SNAP25 (pos = 14):
##
## CSF_ABeta42, CSF_PhosphoTau, CSF_SNAP25, CSF_TotalTau, Diagnosis,
## MMSE, Sex
##
## De følgende objekter er maskerede fra SNAP25 (pos = 15):
##
## CSF_ABeta42, CSF_PhosphoTau, CSF_SNAP25, CSF_TotalTau, Diagnosis,
## MMSE, Sex

pairwise.t.test(CSF_PhosphoTau, Diagnosis, p.adj = "none")

##
## Pairwise comparisons using t tests with pooled SD
##
## data: CSF_PhosphoTau and Diagnosis
##
## AD HC MCI_AD Non-AD-ND
## HC 0.02799 - - -
## MCI_AD 0.20915 0.31205 - -
## Non-AD-ND 0.00042 0.93578 0.15658 -
## Non_ND_D 2.3e-07 0.59407 0.03521 0.49408
##
## P value adjustment method: none

tapply(CSF_PhosphoTau, Diagnosis, mean, na.rm=TRUE)

## AD HC MCI_AD Non-AD-ND Non_ND_D
## 88.55102 54.83333 73.20000 53.50000 46.59459

tapply(CSF_PhosphoTau, Diagnosis, sd, na.rm=TRUE)

## AD HC MCI_AD Non-AD-ND Non_ND_D
## 45.85078 24.24390 23.78048 26.18992 23.86124

# one way ANOVA for SNAP-25 between groups - with post-hoc pairwise t-test:

cohort$Diagnosis <- as.factor(cohort$Diagnosis)

model_snap25 <- aov(log(CSF_SNAP25)~Diagnosis+Age+Sex,data = cohort)

plot(model_snap25)


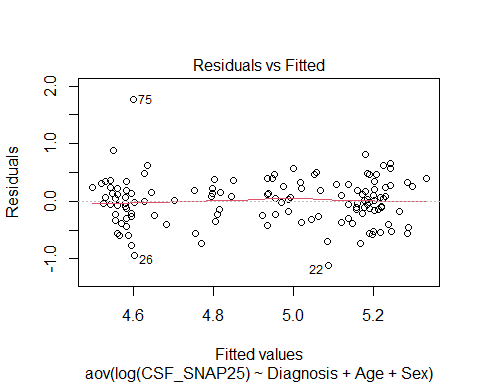

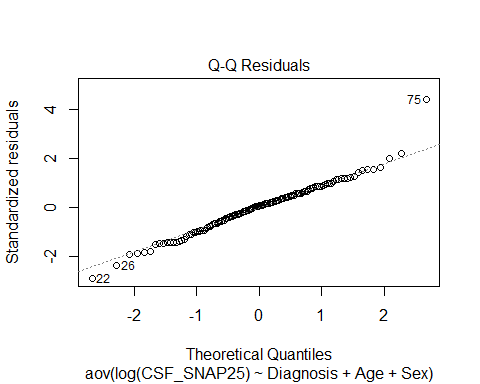

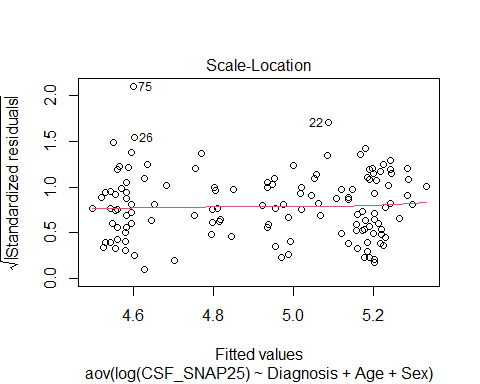

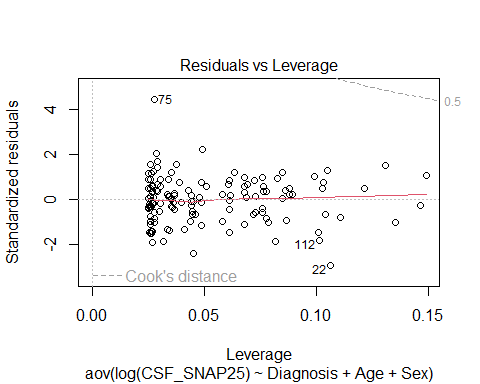


summary(model_snap25)

## Df Sum Sq Mean Sq F value Pr(>F)
## Diagnosis 4 9.470 2.3675 14.370 1.05e-09 ***
## Age 1 0.262 0.2622 1.591 0.209
## Sex 1 0.005 0.0053 0.032 0.858
## Residuals 127 20.923 0.1648
## ---
## Signif. codes: 0 '***' 0.001 '**' 0.01 '*' 0.05 '.' 0.1 ' ' 1

Anova(model_snap25, type = "II")

## Warning in printHypothesis(L, rhs, names(b)): one or more coefficients in the hypothesis include
## arithmetic operators in their names;
## the printed representation of the hypothesis will be omitted

## Anova Table (Type II tests)
##
## Response: log(CSF_SNAP25)
## Sum Sq Df F value Pr(>F)
## Diagnosis 7.9501 4 12.0638 2.428e-08 ***
## Age 0.2652 1 1.6099 0.2068
## Sex 0.0053 1 0.0323 0.8576
## Residuals 20.9234 127
## ---
## Signif. codes: 0 '***' 0.001 '**' 0.01 '*' 0.05 '.' 0.1 ' ' 1

attach(cohort)

## De følgende objekter er maskerede fra cohort (pos = 3):
##
## ...12, AB42_status, Age, CSF_ABeta42, CSF_PhosphoTau, CSF_SNAP25,
## CSF_TotalTau, Diagnosis, Groups, MMSE, Sample ID, Sex
##
## De følgende objekter er maskerede fra cohort (pos = 4):
##
## ...12, AB42_status, Age, CSF_ABeta42, CSF_PhosphoTau, CSF_SNAP25,
## CSF_TotalTau, Diagnosis, Groups, MMSE, Sample ID, Sex
##
## De følgende objekter er maskerede fra cohort (pos = 5):
##
## ...12, AB42_status, Age, CSF_ABeta42, CSF_PhosphoTau, CSF_SNAP25,
## CSF_TotalTau, Diagnosis, Groups, MMSE, Sample ID, Sex
##
## De følgende objekter er maskerede fra cohort (pos = 6):
##
## ...12, AB42_status, Age, CSF_ABeta42, CSF_PhosphoTau, CSF_SNAP25,
## CSF_TotalTau, Diagnosis, Groups, MMSE, Sample ID, Sex
##
## De følgende objekter er maskerede fra SNAP25 (pos = 13):
##
## CSF_ABeta42, CSF_PhosphoTau, CSF_SNAP25, CSF_TotalTau, Diagnosis,
## MMSE, Sex
##
## De følgende objekter er maskerede fra SNAP25 (pos = 14):
##
## CSF_ABeta42, CSF_PhosphoTau, CSF_SNAP25, CSF_TotalTau, Diagnosis,
## MMSE, Sex
##
## De følgende objekter er maskerede fra SNAP25 (pos = 15):
##
## CSF_ABeta42, CSF_PhosphoTau, CSF_SNAP25, CSF_TotalTau, Diagnosis,
## MMSE, Sex
##
## De følgende objekter er maskerede fra SNAP25 (pos = 16):
##
## CSF_ABeta42, CSF_PhosphoTau, CSF_SNAP25, CSF_TotalTau, Diagnosis,
## MMSE, Sex

pairwise.t.test(CSF_SNAP25, Diagnosis, p.adj = "none")

##
## Pairwise comparisons using t tests with pooled SD
##
## data: CSF_SNAP25 and Diagnosis
##
## AD HC MCI_AD Non-AD-ND
## HC 0.0034 - - -
## MCI_AD 0.3763 0.1534 - -
## Non-AD-ND 0.0403 0.3523 0.5078 -
## Non_ND_D 1.1e-07 0.3770 0.0149 0.0333
##
## P value adjustment method: none

tapply(CSF_SNAP25, Diagnosis, mean, na.rm=TRUE)

## AD HC MCI_AD Non-AD-ND Non_ND_D
## 195.4220 130.7643 173.3800 154.6056 111.1571

tapply(CSF_SNAP25, Diagnosis, sd, na.rm=TRUE)

## AD HC MCI_AD Non-AD-ND Non_ND_D
## 74.54158 44.35484 64.77087 50.49080 83.17841

#FURTHER ANALYSIS OF SNAP-25 IN THE PROSPECTIVE STUDY
#Packages:
library(readxl)
library(dplyr)
library(tidyverse)

setwd("P:/RH/NEU/Lukkede Mapper/BiobankCSFProjekt2017/SNAP-25/Statistik_mappe/R/Prospektivt")
cohort <- read_excel('Prospective study_demografitabel.xlsx')

## New names:
## • `` -> `...12`

#FIRST RESEARCH QUESTION - SNAP-25 ABILITY TO DIFFERENTIATE BETWEEN AD DISEASE STAGES
# Division of the cohort in the desired groups - HC, MCI_AD, AD:

cohort$Diagnosis <- as.factor(cohort$Diagnosis)
cohort2 <- cohort[cohort$Diagnosis %in% c('AD', 'MCI_AD', 'HC'),] # Definerende ift. kohorten
cohort2$Diagnosis <- droplevels(cohort2$Diagnosis)

# chi squared test for sex distribution between the predefined groups:
tabel <- table(cohort2$Sex, cohort2$Diagnosis)
tabel

##
## AD HC MCI_AD
## F 31 5 8
## M 19 9 2

chisq.test(tabel)

## Warning in chisq.test(tabel): Chi-squared approximation may be incorrect

##
## Pearson's Chi-squared test
##
## data: tabel
## X-squared = 5.1589, df = 2, p-value = 0.07582

# one way ANOVA for age between the predefined groups:
model_age <- aov(Age~Diagnosis, data = cohort2)
plot(model_age)


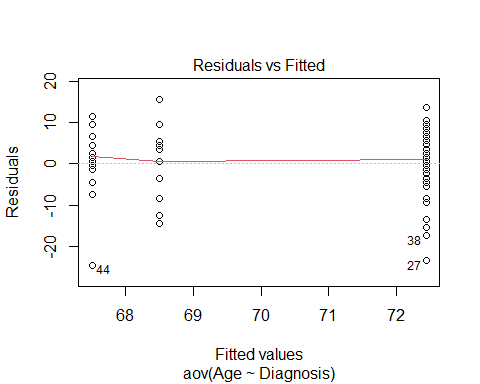

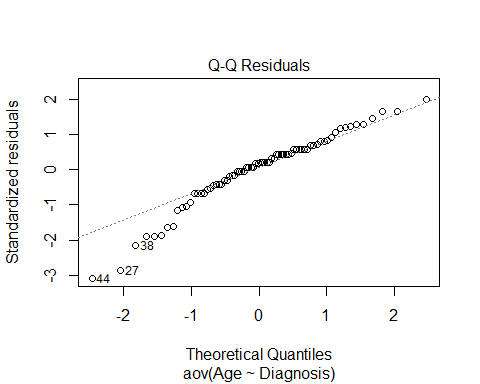

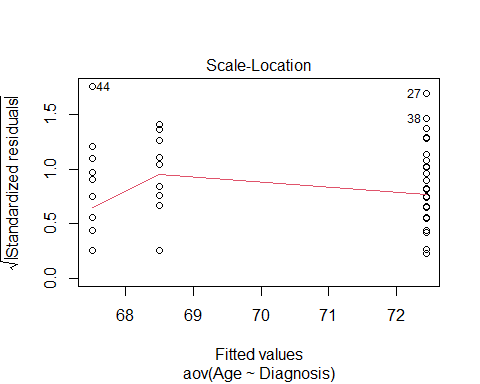

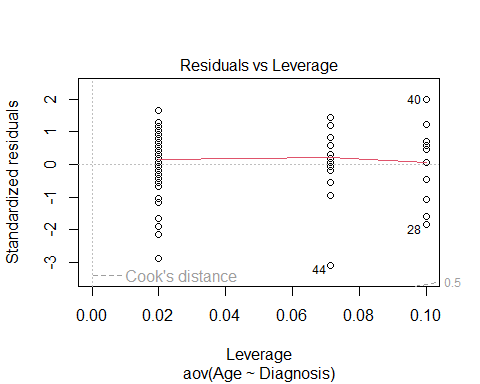


library(car)
Anova(model_age, type = "II") # p-value for the model with a type 2 sum and squares

## Anova Table (Type II tests)
##
## Response: Age
## Sum Sq Df F value Pr(>F)
## Diagnosis 337.6 2 2.4865 0.09042 .
## Residuals 4820.3 71
## ---
## Signif. codes: 0 '***' 0.001 '**' 0.01 '*' 0.05 '.' 0.1 ' ' 1

# an ANCOVA for comparing SNAP-25 between the predefined groups with age as a covariate:

model_SNAP_1 <- aov(log(CSF_SNAP25)~Diagnosis, data = cohort2)
plot(model_SNAP_1) # control of model


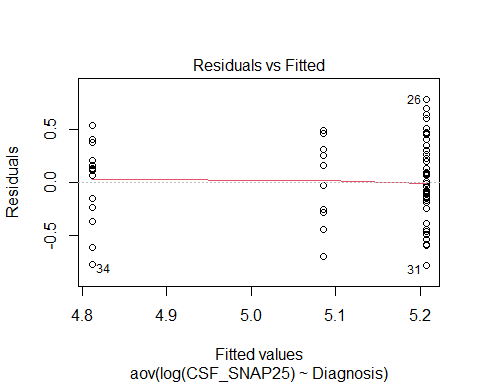

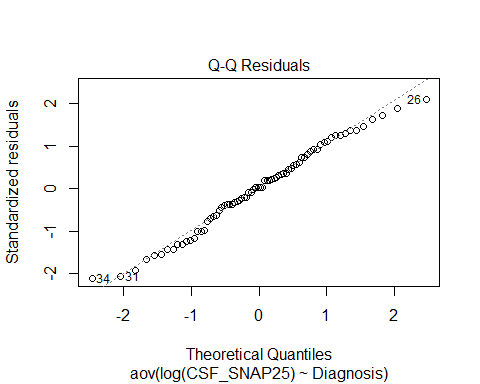

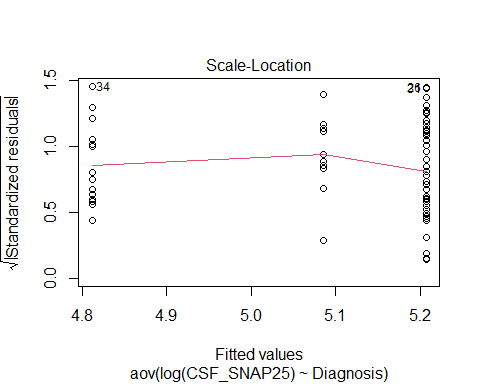

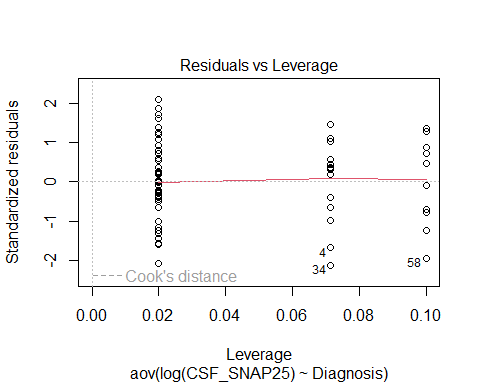


Anova(model_SNAP_1, type = "II")

## Anova Table (Type II tests)
##
## Response: log(CSF_SNAP25)
## Sum Sq Df F value Pr(>F)
## Diagnosis 1.7201 2 6.0266 0.003824 **
## Residuals 10.1325 71
## ---
## Signif. codes: 0 '***' 0.001 '**' 0.01 '*' 0.05 '.' 0.1 ' ' 1

# post-hoc Tukey's test when comparing SNAP-25 between the predefined diagnostic groups:
library(multcomp)
SNAP_mult <- glht(model_SNAP_1, linfct = mcp(Diagnosis = 'Tukey'))
summary(SNAP_mult)

##
## Simultaneous Tests for General Linear Hypotheses
##
## Multiple Comparisons of Means: Tukey Contrasts
##
##
## Fit: aov(formula = log(CSF_SNAP25) ~ Diagnosis, data = cohort2)
##
## Linear Hypotheses:
## Estimate Std. Error t value Pr(>|t|)
## HC - AD == 0 -0.3954 0.1142 -3.461 0.00251 **
## MCI_AD - AD == 0 -0.1211 0.1309 -0.925 0.62057
## MCI_AD - HC == 0 0.2743 0.1564 1.754 0.18849
## ---
## Signif. codes: 0 '***' 0.001 '**' 0.01 '*' 0.05 '.' 0.1 ' ' 1
## (Adjusted p values reported -- single-step method)

#confidensinterval:
confint(SNAP_mult)

##
## Simultaneous Confidence Intervals
##
## Multiple Comparisons of Means: Tukey Contrasts
##
##
## Fit: aov(formula = log(CSF_SNAP25) ~ Diagnosis, data = cohort2)
##
## Quantile = 2.3814
## 95% family-wise confidence level
##
##
## Linear Hypotheses:
## Estimate lwr upr
## HC - AD == 0 -0.39537 -0.66740 -0.12335
## MCI_AD - AD == 0 -0.12110 -0.43275 0.19054
## MCI_AD - HC == 0 0.27427 -0.09821 0.64675

# Bar plot

order_SNAP <- c("HC", "MCI_AD", "AD")
cohort2$Diagnosis <- factor(cohort2$Diagnosis, levels = order_SNAP)
plot(cohort2$CSF_SNAP25~cohort2$Diagnosis)


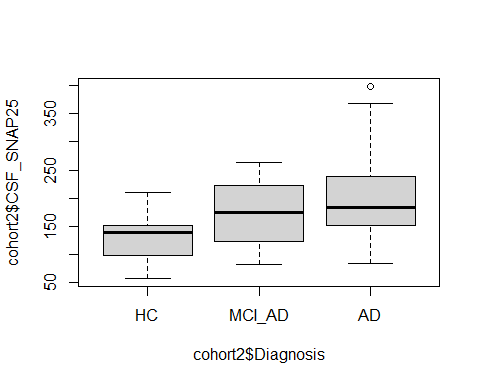


#SECOND RESEARCH QUESTION - SNAP-25 ABILITY TO DIFFERENTIATE BETWEEN PATIENTS WITH OR WITHOUT AD PATHOLOGY
# Division of the cohort in the desired groups - AD Pathology, VaD, NPH:
#1 = AD pathology
#2 = NON-AD-ND
#3 = NON-ND-D

cohort$gruppe <- as.factor(cohort$Groups)

desired_categories <- c("1", "2", "3")
new_dataset_2 <- subset(cohort, cohort$gruppe %in% desired_categories)
new_dataset_2$gruppe <- droplevels(new_dataset_2$gruppe)

# chi squared test for sex distribution between the predefined groups:
tabel <- table(new_dataset_2$Sex, new_dataset_2$gruppe)
tabel

##
## 1 2 3
## F 39 7 10
## M 21 11 32

chisq.test(tabel)

##
## Pearson's Chi-squared test
##
## data: tabel
## X-squared = 17.357, df = 2, p-value = 0.0001702

# one way ANOVA for age between the predefined groups:
model_age_a2 <- aov(Age~gruppe, data = new_dataset_2)
plot(model_age_a2)


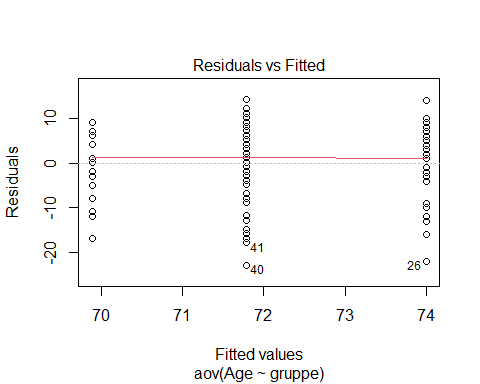

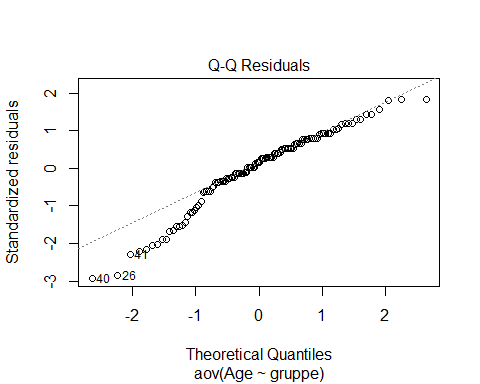

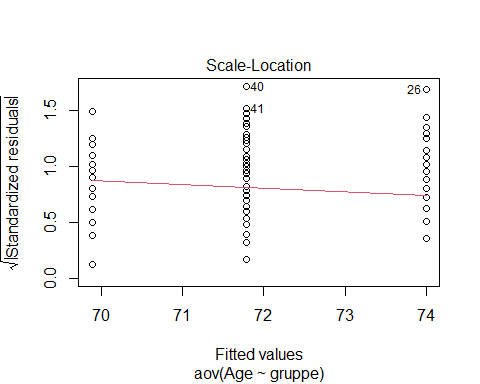

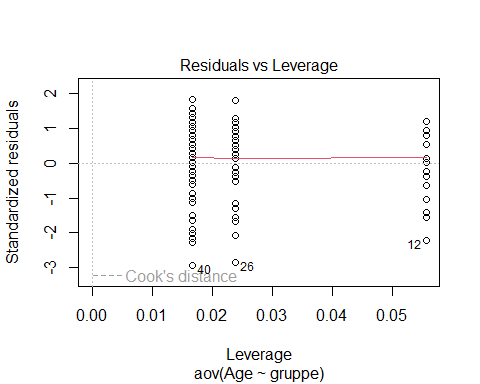


Anova(model_age_a2, type = "II") # p-value for the model with a type 2 sum and squares

## Anova Table (Type II tests)
##
## Response: Age
## Sum Sq Df F value Pr(>F)
## gruppe 242 2 1.9649 0.1448
## Residuals 7204 117

# an ANCOVA for comparing SNAP-25 between the predefined groups with sex distribution as the covariate:
model_SNAP_2 <- aov(log(CSF_SNAP25)~gruppe + Sex, data = new_dataset_2)
plot(model_SNAP_2) # control of model


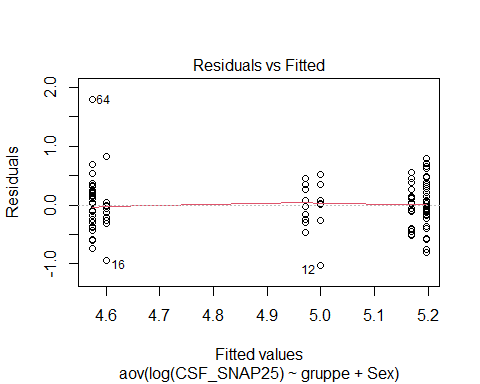

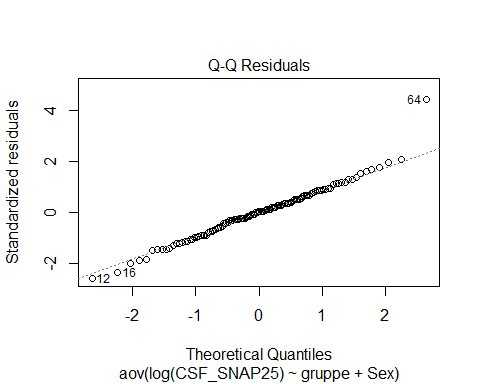

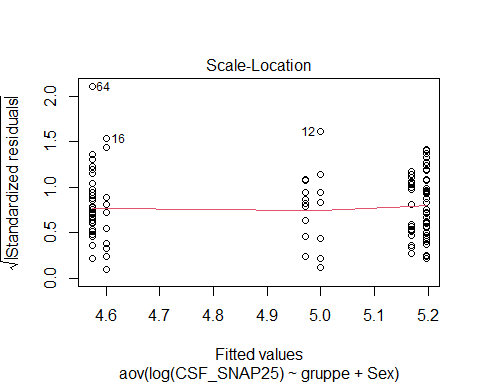

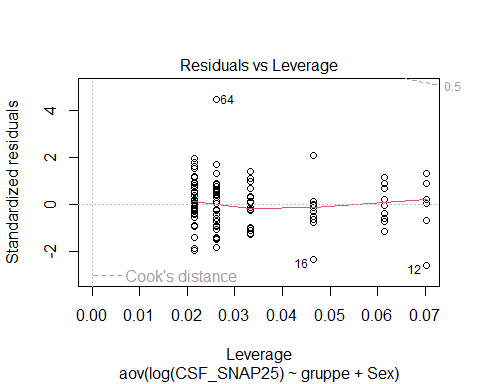


Anova(model_SNAP_2, type = "II")

## Anova Table (Type II tests)
##
## Response: log(CSF_SNAP25)
## Sum Sq Df F value Pr(>F)
## gruppe 7.5971 2 22.7011 4.784e-09 ***
## Sex 0.0188 1 0.1124 0.738
## Residuals 19.4101 116
## ---
## Signif. codes: 0 '***' 0.001 '**' 0.01 '*' 0.05 '.' 0.1 ' ' 1

# Post-hoc Tukey's test comparing SNAP-25 between the predefined groups:
SNAP_mult_2 <- glht(model_SNAP_2, linfct = mcp(gruppe = 'Tukey'))
summary(SNAP_mult_2)

##
## Simultaneous Tests for General Linear Hypotheses
##
## Multiple Comparisons of Means: Tukey Contrasts
##
##
## Fit: aov(formula = log(CSF_SNAP25) ~ gruppe + Sex, data = new_dataset_2)
##
## Linear Hypotheses:
## Estimate Std. Error t value Pr(>|t|)
## 2 - 1 == 0 -0.19810 0.11194 -1.770 0.18223
## 3 - 1 == 0 -0.59569 0.08879 -6.709 < 1e-04 ***
## 3 - 2 == 0 -0.39759 0.11588 -3.431 0.00226 **
## ---
## Signif. codes: 0 '***' 0.001 '**' 0.01 '*' 0.05 '.' 0.1 ' ' 1
## (Adjusted p values reported -- single-step method)

plot(log(new_dataset_2$CSF_SNAP25)~new_dataset_2$gruppe)


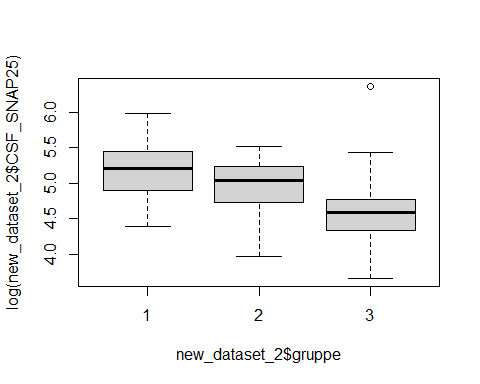


#confidensinterval:
confint(SNAP_mult_2)

##
## Simultaneous Confidence Intervals
##
## Multiple Comparisons of Means: Tukey Contrasts
##
##
## Fit: aov(formula = log(CSF_SNAP25) ~ gruppe + Sex, data = new_dataset_2)
##
## Quantile = 2.368
## 95% family-wise confidence level
##
##
## Linear Hypotheses:
## Estimate lwr upr
## 2 - 1 == 0 -0.19810 -0.46318 0.06699
## 3 - 1 == 0 -0.59569 -0.80595 -0.38543
## 3 - 2 == 0 -0.39759 -0.67200 -0.12318

# ASSOCIATION OF SNAP-25 WITH TAU PROTEINS:
# Linear regression of SNAP-25 and p-tau (WITHOUT LOG)

linear_model <- lm(CSF_SNAP25~CSF_PhosphoTau, data = cohort)
summary(linear_model)

##
## Call:
## lm(formula = CSF_SNAP25 ~ CSF_PhosphoTau, data = cohort)
##
## Residuals:
## Min 1Q Median 3Q Max
## -197.34 -26.68 -11.84 20.64 300.02
##
## Coefficients:
## Estimate Std. Error t value Pr(>|t|)
## (Intercept) 62.630 10.600 5.909 3.42e-08 ***
## CSF_PhosphoTau 1.443 0.136 10.609 < 2e-16 ***
## ---
## Signif. codes: 0 '***' 0.001 '**' 0.01 '*' 0.05 '.' 0.1 ' ' 1
##
## Residual standard error: 58.31 on 118 degrees of freedom
## (14 observations deleted due to missingness)
## Multiple R-squared: 0.4882, Adjusted R-squared: 0.4839
## F-statistic: 112.6 on 1 and 118 DF, p-value: < 2.2e-16

plot(linear_model)


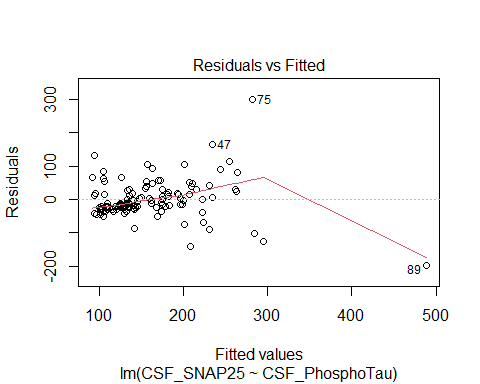

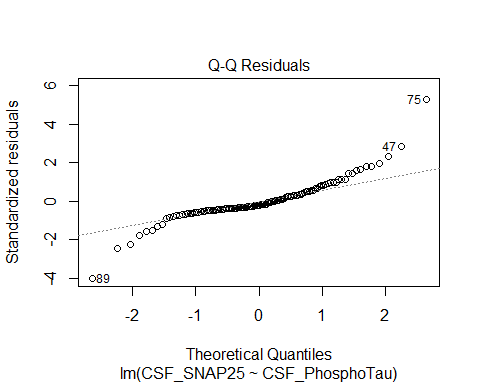

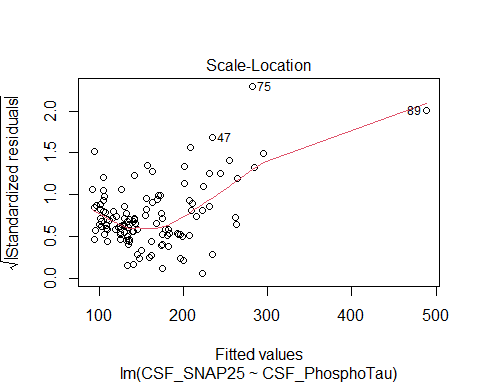

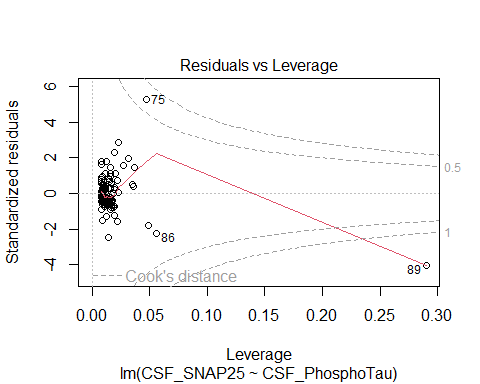


# Linear regression of SNAP-25 and p-tau (WITH LOG)

linear_model_ptau <- lm(log(CSF_SNAP25)~CSF_PhosphoTau, data = cohort)
summary(linear_model_ptau)

##
## Call:
## lm(formula = log(CSF_SNAP25) ~ CSF_PhosphoTau, data = cohort)
##
## Residuals:
## Min 1Q Median 3Q Max
## -1.17888 -0.17282 0.01354 0.19870 0.83887
##
## Coefficients:
## Estimate Std. Error t value Pr(>|t|)
## (Intercept) 4.403464 0.062185 70.81 <2e-16 ***
## CSF_PhosphoTau 0.008301 0.000798 10.40 <2e-16 ***
## ---
## Signif. codes: 0 '***' 0.001 '**' 0.01 '*' 0.05 '.' 0.1 ' ' 1
##
## Residual standard error: 0.3421 on 118 degrees of freedom
## (14 observations deleted due to missingness)
## Multiple R-squared: 0.4784, Adjusted R-squared: 0.474
## F-statistic: 108.2 on 1 and 118 DF, p-value: < 2.2e-16

plot(linear_model_ptau)


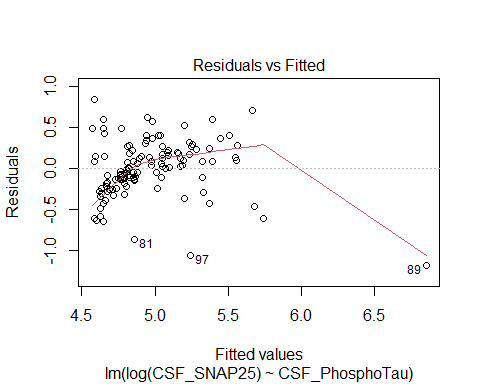

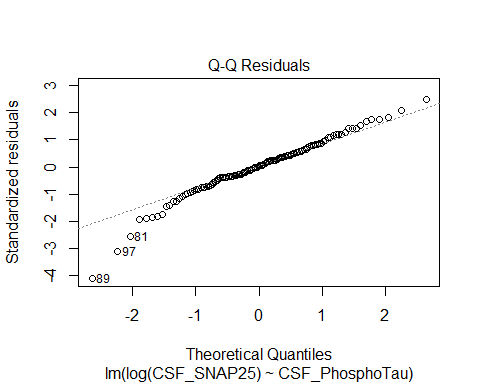


# Linear regression of SNAP-25 and t-tau (WITHOUT LOG)

linear_model_2 <- lm(CSF_SNAP25~CSF_TotalTau, data = cohort)
summary(linear_model_2)

##
## Call:
## lm(formula = CSF_SNAP25 ~ CSF_TotalTau, data = cohort)
##
## Residuals:
## Min 1Q Median 3Q Max
## -146.678 -21.009 -3.511 19.623 228.965
##
## Coefficients:
## Estimate Std. Error t value Pr(>|t|)
## (Intercept) 46.91339 7.30558 6.422 2.77e-09 ***
## CSF_TotalTau 0.25510 0.01436 17.768 < 2e-16 ***
## ---
## Signif. codes: 0 '***' 0.001 '**' 0.01 '*' 0.05 '.' 0.1 ' ' 1
##
## Residual standard error: 42.84 on 121 degrees of freedom
## (11 observations deleted due to missingness)
## Multiple R-squared: 0.7229, Adjusted R-squared: 0.7206
## F-statistic: 315.7 on 1 and 121 DF, p-value: < 2.2e-16

plot(linear_model_2)

# Linear regression of SNAP-25 and t-tau (WITH LOG)

linear_model_ttau <- lm(log(CSF_SNAP25)~CSF_TotalTau, data = cohort)
summary(linear_model_ttau)

##
## Call:
## lm(formula = log(CSF_SNAP25) ~ CSF_TotalTau, data = cohort)
##
## Residuals:
## Min 1Q Median 3Q Max
## -0.8552 -0.1355 0.0131 0.1758 0.5601
##
## Coefficients:
## Estimate Std. Error t value Pr(>|t|)
## (Intercept) 4.305e+00 4.528e-02 95.08 <2e-16 ***
## CSF_TotalTau 1.475e-03 8.898e-05 16.58 <2e-16 ***
## ---
## Signif. codes: 0 '***' 0.001 '**' 0.01 '*' 0.05 '.' 0.1 ' ' 1
##
## Residual standard error: 0.2655 on 121 degrees of freedom
## (11 observations deleted due to missingness)
## Multiple R-squared: 0.6943, Adjusted R-squared: 0.6917
## F-statistic: 274.8 on 1 and 121 DF, p-value: < 2.2e-16

plot(linear_model_ttau)

# Association between SNAP-25 and p-tau
library(ggplot2)
ggplot(cohort, aes(x=CSF_SNAP25, y=CSF_PhosphoTau, color = Diagnosis)) +
 geom_point(size = 3) +
 geom_smooth(method=lm , color="red", fill="#69b3a2", se=TRUE) +
 xlab("CSF SNAP25 [pg/ml]") + ylab("CSF ptau [pg/ml]")

## `geom_smooth()` using formula = 'y ~ x'

## Warning: Removed 14 rows containing non-finite values (`stat_smooth()`).

## Warning: Removed 14 rows containing missing values (`geom_point()`).

# Association between SNAP-25 and t-tau
library(ggplot2)
ggplot(cohort, aes(x=CSF_SNAP25, y=CSF_TotalTau, color = Diagnosis)) +
 geom_point(size = 3) +
 geom_smooth(method=lm , color="red", fill="#69b3a2", se=TRUE) +
 xlab("CSF SNAP25 [pg/ml]") + ylab("CSF Total tau [pg/ml]")

## `geom_smooth()` using formula = 'y ~ x'

## Warning: Removed 11 rows containing non-finite values (`stat_smooth()`).

## Warning: Removed 11 rows containing missing values (`geom_point()`).

# Removed outlier SNAP-25 and p-tau
cohort_outlier <- cohort[!CSF_PhosphoTau>290,]
linear_model_or <- lm(CSF_SNAP25~CSF_PhosphoTau, data = cohort_outlier)
summary(linear_model_or)

##
## Call:
## lm(formula = CSF_SNAP25 ~ CSF_PhosphoTau, data = cohort_outlier)
##
## Residuals:
## Min 1Q Median 3Q Max
## -161.51 -24.05 -10.33 21.83 268.57
##
## Coefficients:
## Estimate Std. Error t value Pr(>|t|)
## (Intercept) 41.745 11.017 3.789 0.00024 ***
## CSF_PhosphoTau 1.787 0.150 11.915 < 2e-16 ***
## ---
## Signif. codes: 0 '***' 0.001 '**' 0.01 '*' 0.05 '.' 0.1 ' ' 1
##
## Residual standard error: 54.41 on 117 degrees of freedom
## (14 observations deleted due to missingness)
## Multiple R-squared: 0.5482, Adjusted R-squared: 0.5443
## F-statistic: 142 on 1 and 117 DF, p-value: < 2.2e-16

plot(linear_model_or)

# Association between SNAP-25 and p-tau
library(ggplot2)
ggplot(cohort_outlier, aes(x=CSF_SNAP25, y=CSF_PhosphoTau, color = Diagnosis)) +
 geom_point(size = 3) +
 geom_smooth(method=lm , color="red", fill="#69b3a2", se=TRUE) +
 xlab("CSF SNAP25 [pg/ml]") + ylab("CSF ptau [pg/ml]")

## `geom_smooth()` using formula = 'y ~ x'

## Warning: Removed 14 rows containing non-finite values (`stat_smooth()`).

## Warning: Removed 14 rows containing missing values (`geom_point()`).
